# Using the situational characteristics of the DIAMONDS taxonomy to distinguish sports to more precisely investigate their relation with psychologically relevant variables

**DOI:** 10.1371/journal.pone.0241013

**Published:** 2020-10-22

**Authors:** Sophia Terwiel, John F. Rauthmann, Maike Luhmann

**Affiliations:** 1 Department of Psychological Methodology, Ruhr University Bochum, Bochum, Germany; 2 Personality Psychology and Psychological Assessment, Universität Bielefeld, Bielefeld, Germany; 3 Department of Psychological Methodology, Ruhr University Bochum, Bochum, Germany; Univeristy of Padova, ITALY

## Abstract

The continuous development and evolvement of sports provide a challenge for researchers who study psychological correlates and consequences of sports, as no single study can include all sports and results cannot easily be generalized across different sports. In this preregistered study, we present a new way of distinguishing sports based on the eight DIAMONDS situational characteristics: Duty, Intellect, Adversity, Mating, pOsitivity, Negativity, Deception, and Sociality. In a cross-sectional online survey, athletes were asked to judge the sport they perform on the eight DIAMONDS dimensions. 138 sports were rated by *N* = 7,835 athletes using the 24-item version of the S8*questionnaire measuring the DIAMONDS. Descriptive and cluster analyses were performed, and situational characteristics profiles were computed. The sport-specific profiles and identified clusters resemble existing sport categorizations but add relevant information based on the situational characteristics of sports, especially regarding their relation with psychologically relevant variables.

## Introduction

Sports are continuously evolving. New or adapted sports like ultimate frisbee or futsal are added to classic sports like soccer or track and field. Some lists enumerate more than 3000 different sports [1]. This large and steadily growing number of different sports provides a challenge for researchers who study psychological correlates and consequences of physical activity. As no single study can include all sports in its sample, it is impossible to know whether effects found for the sport(s) or physical activity examined in one particular study can be generalized to other sports.

A solution to this problem is to group sports into different categories based on medical or physiological characteristics [2,3], external factors such as the required equipment [4] or how athletes compete against each other [5], or social-psychological factors such as the number of people involved and the level of interaction and cooperation needed [6] for an overview of some existing classifications, see Table 1).

**Table 1:** Existing distinctions of sport applied to six different sports.

Category	Rugby	Judo	Swimming	Vaulting	Outdoor Climbing	Chess
Organized vs. Non-organized	Organized	Organized	Can be both	Merely organized	Merely non- organized	Can be both
Social/Team vs. Non-social/ Individual	Team	Individual	Individual	Individual	Individual	Individual
High-risk vs. Low-risk sports	High	Low	Low	Low	High	Low
Type of competition	Sport game	Martial arts	Centimeter-gram-second sport	Compositional sports	Other	No sports
Aerobic vs. Anaerobic	Mixed	Mixed	Aerobic	Anaerobic	Mixed	No sports

In applied sport psychology, these categories are usually sufficient. For example, sport-psychological interventions focus on different abilities depending on whether they are aimed at individuals [7] or teams [8,9]. For psychological research, however, these rather crude categories are of limited utility. Some categories are based on objective cues with little psychological relevance, for example, the type of competition [5]. Moreover, categories that are based on psychological characteristics often focus on one specific psychological aspect and neglect others. As one example, many studies examining the relation between the type of sport and an athlete’s personality distinguish between team sports and individual sports [10–14]. The distinction between team sports and individual sports is also used to explain differences in loneliness [15], mental health [16], or well-being [17] among participants of different sports. One reason for why the distinction between team sports and individual sports is used so frequently might be that it taps into a psychological characteristic that is relevant for these kinds of psychological variables: whether and to what extent the sport involves social interactions.

However, the dichotomous distinction between team sports and individual sports may be too simple. For example, even individual sports such as running may involve social interaction if the training is performed in a group [18]. Hence, sports differ in the *degree* of social interactions and therefore cannot simply be grouped into those that do involve social interactions and those that do not. More generally, dichotomies are inadequate to describe differences in the degree to which a certain characteristic applies to a sport. Additionally, simple dichotomies focus on only one aspect of a sport and necessarily neglect other relevant aspects. For example, the valence of a sport–whether it is perceived positively or negatively while performing it–may influence how strongly a particular sport is associated with psychological variables such as personality or well-being, independent of whether it is a team sport or an individual sport.

To get a better understanding of why different sports differ in their associations with psychological variables, it is necessary to describe similarities and differences among sports in a more systematic and comprehensive way than it has been previously done in the literature. The current paper addresses this gap by conceptualizing sports as standardized situations [19] that can be described on a range of different characteristics for reviews, see [20–23]. The present study presents a new approach to describe and distinguish sports based on psychological situation characteristics as defined in the DIAMONDS taxonomy [24].

### Sports as situations

Sports follow a clear set of rules which define and structure them [25], and also distinguish them from another [19]. Rules structure the situation and allow for a specified and quite limited range of different behavioral options. For example, in soccer, a player receiving the ball can either pass immediately or run with the ball. However, he cannot decide to pick up the ball with his hand, because he would break a rule, resulting in a punishment. Thus, the given rules in a sport lead to clear structure and limited situational characteristics of a given sport. Therefore, we propose that sports can be described and distinguished based on their typical situational characteristics.

Despite its frequent use in everyday life, the definition of the term situation has led to ongoing discussions [26] and should not be issued in the current work. To still allow systematic and generalizable research on how situations are linked to psychological processes, we will focus on the widely accepted use of terminology regarding the classification of situations based on features such as classes, cues, or situation characteristics [23]. Situational classes distinguish between life domains, for example, work situations or academic situations. Situational classes are not useful to distinguish between sports because most sports would fall into the same class. Situational cues distinguish situations based on objective, constituent elements such as weather or the presence of an audience. Most existing classifications of sports use situational cues, for example, on the use of specific equipment [27]. Finally, classifications focusing on situational characteristics distinguish situations based on psychologically meaningful attributes describing these situations. We argue that situational characteristics may be particularly useful to distinguish and classify sports when investigating differences and similarities between the relation of participation in different sports and psychologically relevant variables. We argue that situational characteristics (e.g. sociality) are more informative than classes (e.g. sport situation) and more relevant than cues (e.g. use of equipment).

One widely used classification using situation characteristics is the DIAMONDS taxonomy [24,28] which comprises eight continuous dimensions: (1) Duty (situation contains tasks and work), (2) Intellect (situation contains intellectual engagement), (3) Adversity (situation contains threats, problems, competition, or criticism), (4) Mating (situation contains sex, love, and romance), (5) pOsitivity (situation may be pleasant and joyful), (6) Negativity (situation could elicit negative feelings like frustration or anxiety), (7) Deception (situation contains mistrust, lying, or betrayal) and (8) Sociality (situation contains socializing, relationship formation, or communication).

The DIAMONDS taxonomy has been used to shed light on different psychological research questions. For example, Sherman, Rauthmann, Brown, Serfass, and Jones [29] found in an experience sampling study that situational as well as personality characteristics explained differences in real-time emotions and behavior. The reciprocal relationship between personality and situational characteristics has also been investigated in other studies [30–33]. The findings from these studies show that the DIAMONDS taxonomy adds important information when investigating relations between psychological variables such as personality or emotions. The DIAMONDS taxonomy has also been applied to the processes of personnel selection and development. However, to our knowledge, the DIAMONDS taxonomy has not yet been applied in the field of sport psychology even though situational aspects are of high relevance in the context of sports. For example, the DIAMONDS dimension of deception might be more relevant in sports that include the legal deceptive behavior of athletes such as feinted movements or plays (e.g., ball games, fighting sports) [34]. For most sports, however, it is not possible to make specific a priori predictions about how they are related to each of the DIAMONDS dimensions. For this reason, we adopted a purely exploratory approach to investigate the associations between sports and the DIAMONDS.

### The present study

The goal of the present study was use the psychological situation characteristics measured via the DIAMONDS [24] to identify differences and similarities among sports. First, we examined whether the mean scores of the DIAMONDS dimensions differed among different sports. Second, we investigated whether these differences could be used to distinguish sports based on their situational profile patterns. Lastly, we examined whether meaningful clusters of similar sports could be identified in cluster analyses. For this purpose, we conducted an online survey in which athletes of different sports rated the situational characteristics of their sports using the DIAMONDS taxonomy.

## Materials and methods

### Procedure and sample

An English and a German version of a 20-minute online survey were provided with the survey tool Qualtrics [35]. Athletes of different sports rated the situational characteristics of the sport they performed most frequently. Participants were recruited via sport-specific groups on Facebook and Reddit, online forums, or personal contact. To compensate for their participation, they received feedback on the typical personality profile of athletes of their sport via email. The data collection was conducted with the approval of the local ethics committee of the Ruhr University Bochum, which follows the recommendations of The German Psychological Society (DGPs). To take part in the survey, participants had to confirm that they were older than 18 years and they had to provide informed consent.

The final sample was determined via the following steps. First, participants with missing data on either the question for their main sport or the item assessing situational characteristics were excluded. Second, participants who did not provide a sport as main sport that we could clearly assign to a sport category (*N* = 248) were excluded (see below for details on how the main sport was determined for each participant). Third, we excluded entire sport categories if there was only one rater (17 sports) or if the intraclass correlation reflecting the agreement among all raters of this sport (see below) was less than .75 (11 sports), the cutoff for excellent interrater agreement according to Cicchetti [36] (see S1 Table). The final sample thus comprised *N* = 7,835 athletes (32.45% female, 67.37% male, 0.18% other gender) rating 138 sports (for a list of all sports included in the data collection, see S2 Table), with an average number of 56.78 (range: 4–263) raters per sport.

### Measures

The data analyzed for the present study were collected as part of a large-scale survey on sports, athlete’s personality, situational aspects of sport, and the relation between sport and different variables. In addition, questions on demographics and sports (frequency, level of performance, motives for participation), affect, life satisfaction, and the valuing of happiness and loneliness were administered. Moreover, expert ratings for the typical and ideal athlete’s personalities of the most frequently performed sport were requested. For more detailed information on the questionnaires used, see the preregistration of the data collection (https://osf.io/m9nc5/?view_only=3040c7b92193456d8e511dae4777e41f).

### Sport

We created a list of 167 sports by combining literature research and online research (e.g. International Olympic Committee). The included sports were popular sports commonly practiced in those countries in which we had planned to recruit study participants. Participants selected the sport they perform most frequently out of this list of 167 different categories, which also included the option “other than stated.” They also had the opportunity to name their sport in an open-format question. These open responses were manually coded by two independent raters. Cohen’s kappa for these codings was .98 and thus excellent. If the two raters chose different categories for a specific case, the senior researcher’s rating was favored. Seven new categories were added because responses could not be clearly assigned to the given categories or because sample sizes for a particular broadly defined sport were large enough to split this sport into different subtypes (for details see S2 Table).

#### Situational characteristics

The instruction of the S8* was adapted such that it referred to the *typical* sport situation perceived by the athletes (“Please indicate for each question to what extent it applies to your sport.”). Moreover, the item ‘A job needs to be done’ of the Duty scale was adapted to ‘A task needs to be done’ to better fit the sport context. Each item used a scale from 1 (*not at all*) to 7 (*totally*). Responses were averaged within each subscale to retrieve aggregated scale scores. Note, however, that some analyses were conducted on the item level (see below) to glean a more differentiated picture and provide more nuance to our findings. Internal consistencies (computed across all sports and raters) were satisfactory considering that 4-item scales were used: Duty (α = .70), Intellect (α = .81), Adversity (α = .60), Mating (α = .59), pOsitivity (α = .67), Negativity (α = .78), Deception (α = .78), Sociality (α = .77).

### Analyses

All statistical analyses were performed in R [37]. Some of the preregistered analyses were dropped due to an adaptation of the theoretical focus of the present paper (For a detailed overview of the planned analyses, see the preregistration: https://osf.io/r3wy9/?view_only=7ea74bae783c41a3baaae2aa9ba62392. One K-means cluster analysis and the analyses of the additional sports performed were not dropped from the final analyses.)

To examine the extent to which athletes of the same sport agreed on their ratings, we estimated the interrater agreement within each sport. The intraclass correlation coefficient (ICC[3, *k*]) was calculated as a measure of overall rater agreement [38] across the 24 S8* items using the ICC function included in the R package “psych” [39]. Because some analyses were performed at the item level (see below), interrater agreement was also computed for each single item using a consensus measure for Likert-scale items [40] available in the R package “consr” [41]. A consensus value of 0 reflects total dissention, whereas a value of 1 reflects total consensus [40].

For all sports included in the data analyses, descriptive statistics such as means, and standard deviations were calculated for all items as well as for the mean scores of the eight DIAMONDS subscales. Additionally, situational profile plots displaying the mean scores for the eight subscales were created of each sport.

To examine whether sports could be meaningfully distinguished based on their situational characteristics, we performed cluster analyses using a two-step procedure. First, a hierarchical agglomerative cluster analysis was used to identify micro-clusters and the appropriate range for the number of clusters. Second, a K-means cluster analysis was used to get a cluster solution in which all sports were assigned to a homogenous cluster [42]. Both cluster analyses were based on the 24 items of the S8* rather than on aggregated scale scores for the eight DIAMONDS dimensions. This was due to two reasons. First, eight variables are not enough for cluster analyses, and second, the chosen procedure additionally allowed the examination of detailed aspects of the dimensions in later steps of the analysis.

Numerous methods and similarity measures are provided for hierarchical agglomerative cluster analyses [43]. To determine which of them would lead to an interpretable and plausible result for our research question, we used a two-step procedure to determine the methods and measures. First, advantages and disadvantages of given measures and methods were compared and contrasted. Second, we analyzed the data of 10 sports with relatively high ICC(3, *k*) values (> .96) and at least 30 raters each. These sports were chosen because it was a priori relatively plausible how they might cluster, that is, we could expect a face-valid clustering. For a more detailed description of the measures and methods included and the results, see https://osf.io/r3wy9/?view_only=7ea74bae783c41a3baaae2aa9ba62392.

Based on the visual inspection and the contextual reasonableness of our pre-analysis, a hierarchical agglomerative cluster analysis using average linkage as the clustering method and correlations as the similarity measure was chosen. The hierarchical agglomerative cluster analysis was performed using the package “pvclust” [44]. Details on the analysis and results are reported in the (S2 Fig).

For the K-means cluster analysis, a two-step procedure was used. First, a range of the number of clusters was determined using visual inspection of the hierarchical dendrogram of the agglomerative hierarchical cluster analysis. Second, the composition and number of these clusters were determined by K-means cluster analysis, using the robust version of K-means cluster analysis from the pamk function in the package “fpc” [45].

## Results

### Consensus

To estimate the reliability of the ratings at the item level, the consensus among raters across all sports was calculated for each of the 24 S8* items (see Table 2). The mean consensus estimates ranged between .47 and .60.

**Table 2:** Average consensus, standard deviations, and range for the 24 S8* items, aggregated across all sports.

Item	*M*	*SD*	Range	Scale
A task needs to be done.	.53	.10	.14 - .71	Duty
Task-oriented thinking is required.	.57	.11	.08 - .77	Duty
I have to fulfill my duties	.53	.10	.00 - .74	Duty
The situation contains intellectual stimuli.	.50	.12	.00 - .74	Intellect
There is the opportunity to demonstrate intellectual capacities.	.50	.10	.20 - .70	Intellect
Information needs to be deeply processed.	.53	.09	.08 - .71	Intellect
I am being criticized.	.47	.13	.00 - .75	Adversity
I am being blamed for something.	.51	.11	.19 - .80	Adversity
I am being threatened by someone or something.	.50	.11	.00 - .70	Adversity
Potential sexual or romantic partners are present.	.53	.07	.32 - .73	Mating
Physical attractiveness is relevant.	.60	.15	.01 - .88	Mating
The situation is sexually charged.	.51	.11	.15 - .78	Mating
The situation is pleasant.	.51	.09	.13 - .74	pOsitivity
The situation is playful.	.57	.13	.17 - .78	pOsitivity
The situation is joyous and exuberant.	.49	.09	.17 - .70	pOsitivity
The situation could elicit stress.	.51	.09	.00 - .76	Negativity
The situation could elicit feelings of tension.	.54	.10	.23 - .72	Negativity
The situation could entail frustration.	.54	.10	.13 - .74	Negativity
It is possible to deceive someone.	.50	.11	.19 - .71	Deception
Someone in this situation could be deceptive.	.52	.17	.01 - .85	Deception
Not dealing with others in an honest way is possible.	.53	.09	.26 - .69	Deception
Close personal relationships are important or can develop.	.58	.15	.11 - .88	Sociality
Others show many communicative signals.	.53	.10	.05 - .70	Sociality
Communication with other people is important or desired.	.52	.12	.17 –.82	Sociality

### Descriptive statistics and profile plots

For the 20 sports with the highest and lowest scores, respectively, descriptive statistics for the eight DIAMONDS dimensions are reported in Table 3 (for a complete list of all sports, see S3 Table. The open-access data analysis script can be used to calculate mean values and standard deviations for each sport on an item base, see: https://osf.io/r3wy9/?view_only=7ea74bae783c41a3baaae2aa9ba6239).

**Table 3:** Mean Values (M) and Standard Deviations (SD) for the 20 highest and 20 lowest sport scores for each of the eight DIAMONDS scales.

20 highest scores																							
	Duty			Intellect			Adversity			Mating			pOsitivity			Negativity			Deception			Sociality	
Sport	*M*	*SD*	Sport	*M*	*SD*	Sport	*M*	*SD*	Sport	*M*	*SD*	Sport	*M*	*SD*	Sport	*M*	*SD*	Sport	*M*	*SD*	Sport	*M*	*SD*
Canyoning	6.48	0.50	Chess	6.64	0.73	eSports	4.38	1.30	Partner dance	4.85	1.34	Capoeira	6.49	0.71	Auto racing	6.37	0.78	Capoeira	5.81	1.17	Touch & Tag rugby	6.48	0.54
Polo	6.39	1.18	Cue sports	6.33	1.01	Wrestling	4.16	1.47	Bodybuilding	4.41	1.62	Parkour	6.33	0.72	Cue sports	6.21	0.69	Dodgeball & Prisonball	5.16	1.28	Polo	6.44	0.54
Gridiron football	6.23	0.76	Brazilian jiu-jitsu	6.04	1.08	Gridiron football	3.92	1.17	Dancing	4.20	1.84	Surfing	6.21	0.89	Wrestling	6.12	0.94	Paintball & Airsoft	4.74	1.43	Capoeira	6.43	0.60
Air sports (planes)	6.07	0.72	Historical European martial arts	5.82	1.27	Kendo	3.89	1.22	Beach volleyball	4.16	1.30	Longboarding	6.20	0.96	eSports	5.91	1.06	Fencing	4.62	1.57	Roller derby	6.39	0.65
Auto racing	6.05	1.05	Swordsmanship	5.79	0.88	Dodgeball & Prisonball	3.80	1.07	Contemporary dance	4.08	1.79	Belly dance	6.20	0.76	Table football	5.90	0.86	Historical European martial arts	4.57	1.66	Dodgeball & Prisonball	6.33	0.50
Australian football & Gaelic football	5.98	0.83	Jiujitsu	5.77	1.46	Ice hockey	3.72	1.36	Cheerleading	3.94	1.24	Partner dance	6.15	0.93	Cricket	5.88	1.47	Auto racing	4.56	1.97	Rugby	6.27	0.77
Baseball & Softball	5.94	1.24	Air hockey	5.75	1.17	Cricket	3.70	1.51	Figure skating	3.79	1.42	Snowboarding & Sandboarding	6.15	0.89	Brazilian jiu-jitsu	5.87	1.05	Badminton	4.54	1.36	Partner dance	6.24	0.87
Cheerleading	5.89	0.75	Tai chi	5.70	1.32	Boxing	3.64	1.35	Ballet	3.73	1.39	Touch & Tag rugby	6.14	0.74	Golf	5.86	1.28	Tennis	4.43	1.75	Curling	6.21	1.13
eSports	5.84	1.16	Air sports (planes)	5.67	1.30	Water polo	3.62	1.36	Health club training	3.62	1.47	Skydiving	6.07	0.85	Chess	5.76	1.23	Wrestling	4.40	1.70	Lacrosse	6.21	0.80
Rowing	5.84	1.00	Curling	5.67	1.42	Australian football & Gaelic football	3.62	1.81	Sprinting	3.49	1.72	Polo	6.06	0.85	Dodgeball & Prisonball	5.76	0.84	Jugger	4.32	1.33	Quidditch	6.19	0.80
Flag football	5.82	1.24	Capoeira	5.58	1.22	Contemporary dance	3.47	1.42	Belly dance	3.43	1.44	Inline skating	6.03	0.90	Air sports (planes)	5.70	1.22	Table football	4.28	1.42	Volleyball	6.16	0.72
Cricket	5.82	0.98	Auto racing	5.54	1.38	Fighting sport—Grappling (Other)	3.41	1.33	Water skiing	3.37	1.81	Windsurfing	6.02	0.94	Figure skating	5.70	1.13	Kickboxing	4.26	1.66	Jugger	6.15	0.68
Rugby	5.79	1.02	Cricket	5.47	1.24	Krav Maga	3.40	1.43	CrossFit	3.36	1.30	Skateboarding	6.00	1.03	Jiujitsu	5.67	1.37	Squash & Racquetball	4.25	1.58	Ultimate	6.06	0.84
Curling	5.79	1.25	Pole vault	5.42	1.11	Historical European martial arts	3.40	1.15	Capoeira	3.31	1.19	Kiteboarding	5.99	0.85	Kendo	5.61	1.14	Cricket	4.20	1.18	Australian football & Gaelic football	6.05	0.68
Sailing	5.77	1.35	eSports	5.41	1.28	Brazilian jiu-jitsu	3.37	1.32	Touch & Tag rugby	3.29	1.08	Zumba	5.98	0.89	Air hockey	5.58	1.29	eSports	4.17	1.49	Handball	5.99	0.79
Synchronized swimming	5.77	1.12	Fencing	5.38	1.27	Hurling & Shinty	3.36	1.49	Swimming	3.28	1.57	Pole dance	5.93	0.97	Roller derby	5.55	1.14	Swordsmanship	4.14	1.83	Equestrian vaulting	5.98	1.16
Lacrosse	5.70	1.15	Mixed martial arts	5.35	1.21	Rowing	3.30	1.32	Trampolining	3.15	1.71	Dancing	5.90	0.99	Ice hockey	5.54	1.16	Boxing	4.12	1.59	Synchronized swimming	5.97	0.95
Hurling & Shinty	5.67	0.91	Kung fu	5.27	1.10	Soccer	3.27	1.49	Bouldering	3.15	1.20	Cue sports	5.83	0.73	Track cycling	5.54	0.89	Flag football	4.12	1.44	Contemporary dance	5.92	1.24
Horseback riding	5.66	1.10	Fighting sport—Grappling (Other)	5.23	0.91	Figure skating	3.27	1.32	Quidditch	3.13	1.13	Alpine skiing	5.83	1.29	Climbing (outdoor)	5.54	1.35	Jiujitsu	4.11	1.61	Gridiron football	5.89	1.05
Underwater hockey	5.65	0.81	Golf	5.14	1.20	Cue sports	3.25	1.99	Breakdancing	3.10	1.43	Dodgeball & Prisonball	5.80	0.84	Historical European martial arts	5.53	1.21	Road bicycle racing	4.10	1.77	Cheerleading	5.89	1.07
20 lowest scores																							
	Duty			Intellect			Adversity			Mating			pOsitivity			Negativity			Deception			Sociality	
Sport	*M*	*SD*	Sport	*M*	*SD*	Sport	*M*	*SD*	Sport	*M*	*SD*	Sport	*M*	*SD*	Sport	*M*	*SD*	Sport	*M*	*SD*	Sport	*M*	*SD*
Inline skating	2.58	1.54	Health club training	2.40	1.38	Pilates	1.19	0.33	Air sports (planes)	1.44	0.67	Health club training	3.50	1.04	Qigong	2.28	1.25	Tae Bo	1.45	0.45	Qigong	2.56	1.11
Yoga	2.68	1.63	Indoor cycling	2.40	1.20	Yoga	1.28	0.57	Darts	1.48	0.87	Wrestling	3.58	1.28	Zumba	2.40	1.60	Inline skating	1.70	1.16	Pilates	2.86	1.69
Longboarding	2.80	1.39	Running	2.44	1.38	Indoor cycling	1.37	0.68	Sport fishing	1.49	0.90	Krav Maga	3.81	1.89	Indoor cycling	2.53	1.46	Slacklining	1.71	1.13	Chess	2.88	1.33
Surfing	2.82	1.31	Pilates	2.48	1.67	Obstacle racing	1.41	0.65	Wrestling	1.57	0.86	Bodybuilding	3.84	1.43	Yoga	2.60	1.20	Canyoning	1.74	0.92	Yoga	2.98	1.62
Windsurfing	2.92	1.53	(Half-) Marathon	2.60	1.40	Trailrunning	1.49	0.79	eSports	1.58	0.94	Rowing	3.89	1.38	Tae Bo	2.76	1.38	Windsurfing	1.78	1.02	Bodyweight exercises	3.05	1.66
Skateboarding	2.95	1.40	Obstacle racing	2.71	1.45	Running	1.49	0.78	Racewalking	1.58	0.56	Triathlon	4.04	1.27	Tai chi	2.93	1.28	Dragon boat	1.79	0.70	Running	3.06	1.54
Zumba	2.96	1.76	Inline skating	2.73	1.50	(Half-) Marathon	1.56	0.81	Chess	1.59	1.02	Kendo	4.30	1.36	Health club training	3.03	1.45	Freediving	1.81	0.94	Health club training	3.29	1.46
Snowboarding & Sandboarding	3.14	1.60	Triathlon	2.83	1.48	Qigong	1.56	0.62	Gridiron football	1.64	0.77	Gridiron football	4.33	0.99	Calisthenics	3.35	1.42	Zumba	1.84	1.40	(Half-) Marathon	3.32	1.49
Qigong	3.22	2.08	Bodyweight exercises	2.86	1.60	Cross-country skiing	1.56	0.74	Paintball & Airsoft	1.64	0.82	Weightlifting	4.35	1.35	Running	3.46	1.57	Cross-country skiing	1.85	0.76	Bodybuilding	3.32	1.38
BMX	3.36	1.45	Zumba	2.96	1.79	Disc golf	1.60	0.70	Disc golf	1.65	0.75	Running	4.35	1.26	Cheerleading	3.50	1.99	Pilates	1.86	1.09	Calisthenics	3.37	1.65
Slacklining	3.49	1.43	Trailrunning	3.03	1.49	Calisthenics	1.60	0.81	Qigong	1.67	0.84	Fighting sport—Grappling (Other)	4.38	0.99	Pole dance	3.51	1.37	Equestrian vaulting	1.86	0.92	Triathlon	3.47	1.54
Kiteboarding	3.49	1.69	Dragon boat	3.08	1.22	Pole dance	1.63	0.68	Shooting sport	1.68	0.96	(Half-) Marathon	4.39	1.16	Inline skating	3.55	1.50	Pole dance	1.88	1.03	Motocross	3.53	1.45
Pole dance	3.53	1.48	Windsurfing	3.11	1.34	Bouldering	1.64	0.73	Ice hockey	1.73	1.01	Boxing	4.40	1.50	Pilates	3.57	1.67	Yoga	1.89	0.93	Zumba	3.58	1.76
Recreational cycling	3.55	1.56	CrossFit	3.13	1.39	Health club training	1.64	0.95	Auto racing	1.73	0.88	Chess	4.43	1.31	Bodyweight exercises	3.68	1.65	Bouldering	1.92	1.05	Weightlifting	3.59	1.58
Cross-country cycling & Mountain biking	3.62	1.50	Cross-country cycling & Mountain biking	3.17	1.31	Zumba	1.65	1.13	Table tennis	1.74	0.80	Air sports (planes)	4.44	1.17	Belly dance	3.69	1.27	Track and field (throwing)	1.94	0.94	Trailrunning	3.62	1.51
Running	3.67	1.67	Tae Bo	3.18	1.32	Bodyweight exercises	1.66	0.82	Australian football & Gaelic football	1.74	1.04	Racewalking	4.46	1.47	Dragon boat	3.73	1.54	Running	1.98	1.15	Sport fishing	3.67	1.50

**Fig 1:**
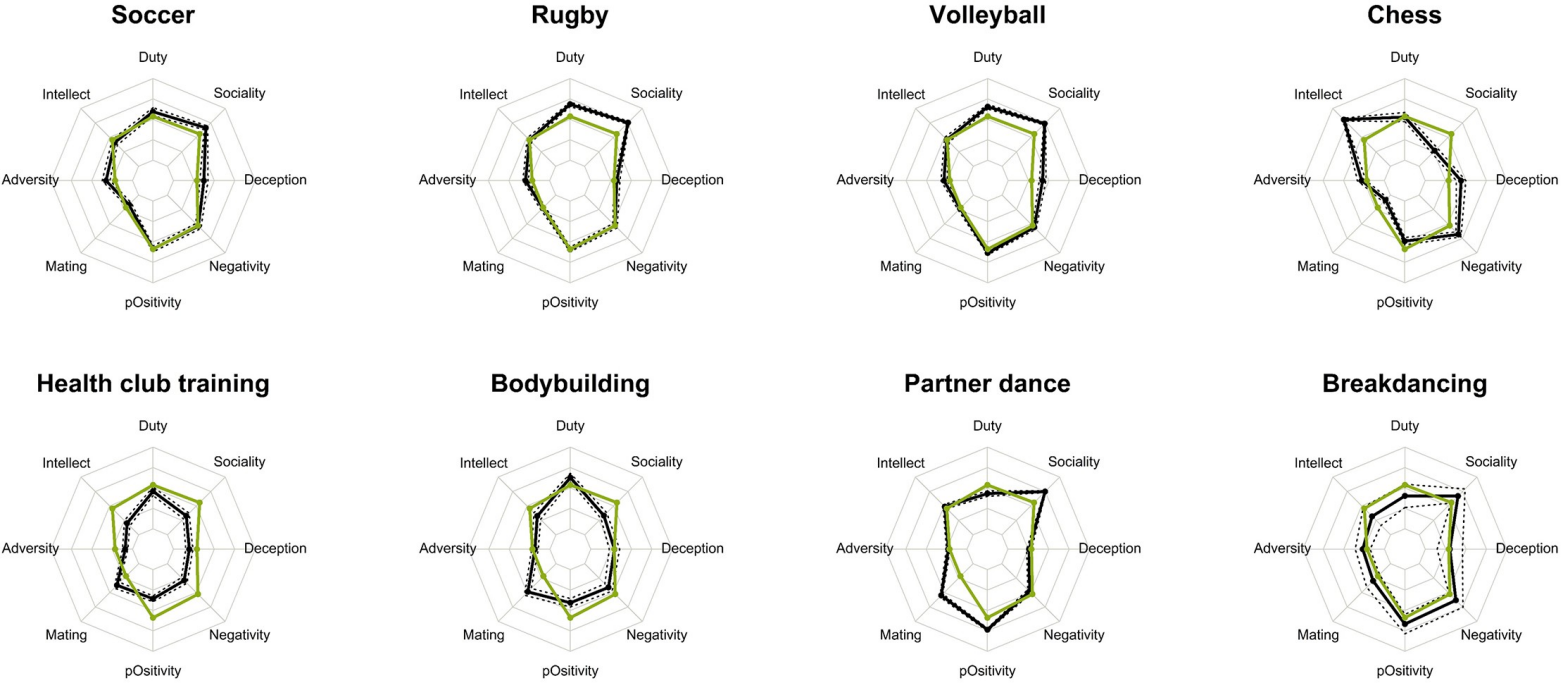
Mean values and standard errors for each of the eight DIAMONDS scales for exemplary sports.

Fig 1 shows examples for situation profiles for eight selected sports (for similar plots for all sports, see S1 Fig) as well as the average values for each dimension. For these sports, the profiles varied in a plausible way, indicating that the situational DIAMONDS can be applied to sports in a meaningful way. For example, partner dance and volleyball scored high on Sociality, whereas chess scored low on Sociality and high on Intellect.

The inner-most line of the web reflects a mean value of 1 and the outer-moist line a value of 7, corresponding to the theoretical minimum and maximum of the response scale used. The values for the situational characteristics of the sports are depicted in black. The values for the mean sport situations for the S8* are depicted in green. Standard errors are depicted in dotted lines.

### Cluster analysis

**Fig 2:**
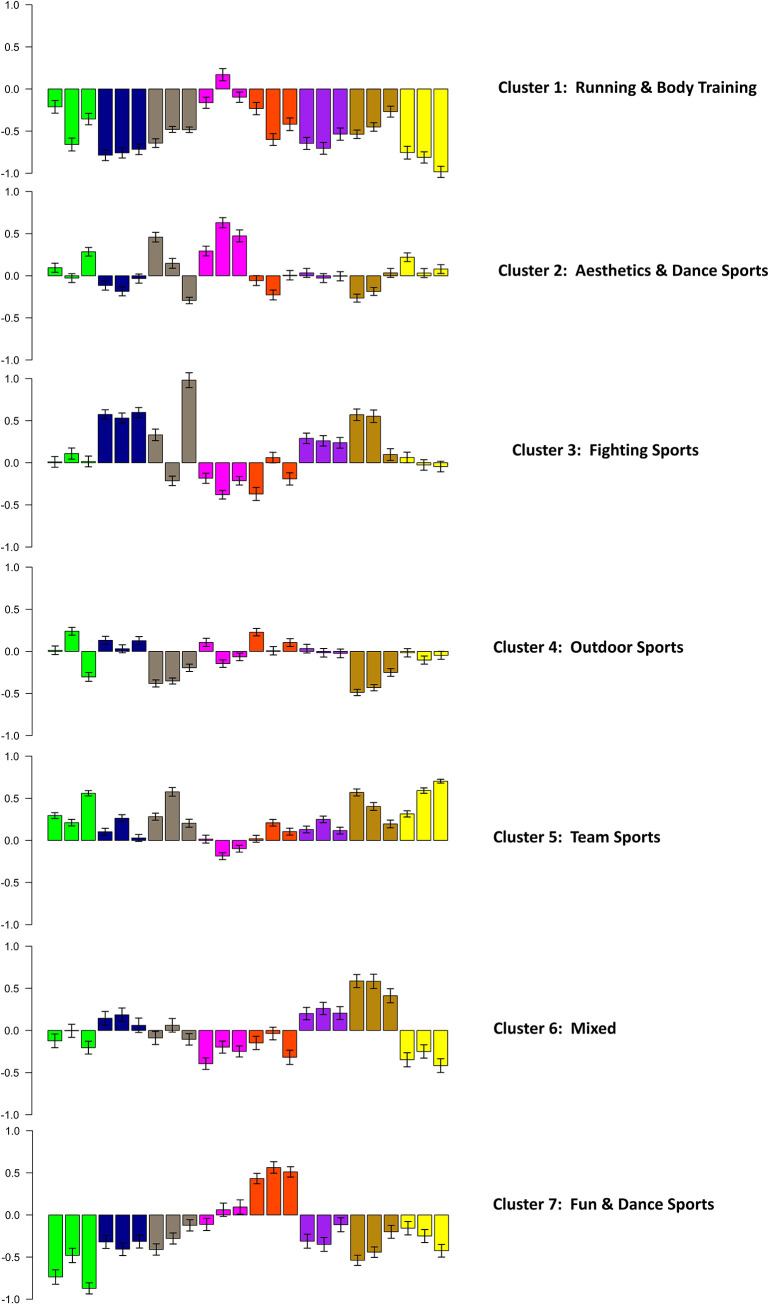
Situational profiles based on the 24 items of S8* for the seven K-means clusters.

Cluster analyses were used to examine whether meaningful clusters of similar sports could be identified, with the criterion of similarity being shared profiles of levels of situation characteristics. First, in a hierarchical cluster analysis, 16 clusters were identified. These clusters included 118 out of 138 sports (see S2 Fig and S4 Table). Second, a K-means cluster analysis was performed, in which each sport was assigned to one cluster. First, we identified between 6 to 16 different clusters via visual inspection of the hierarchical cluster analysis results. Second, an optimal cluster solution was calculated and identified seven clusters (silhouette = .155, indicating no substantial structure). The situational profiles for the seven clusters are depicted in Fig 2. Clusters varied in their level of the situational characteristic at the item level as well as at the scale level.

z-scores are given (range: +1.0 to -1.0) on the y-axis. The three items from the same DIAMONDS scale are depicted in the same pattern. The DIAMONDS scales are depicted in the following order (1) Duty, (2) Intellect, (3) Adversity, (4) Mating, (5) pOsitivity, (6) Negativity, (7) Deception and (8) Sociality.

Cluster 1 (*Running & Body Training*) included sports such as trail running and bodyweight exercises (for assignments of all sports to clusters, see S4 Table) and was characterized by low values on all situational characteristics, especially low Sociality. Cluster 2 (*Aesthetics & Dance Sports*) included sports such as dancing and artistic gymnastics and was characterized by high Sociality and Mating. Cluster 3 (*Fighting Sports*) included sports such as boxing and karate and was characterized by high Negativity, Deception and Intellect. Cluster 4 (*Outdoor Sports*) included sports such as outdoor climbing and sailing and was characterized by low Deception and Adversity. Cluster 5 (*Team Sports*) included sports such as soccer and ice hockey and was characterized by high Sociality, Deception and Duty. Cluster 6 (*Mixed*) included various sports such as table tennis, darts, and track cycling and was characterized by low Mating and Sociality. Cluster 7 (*Fun & Dance Sports*) included sports such as surfing and Zumba and was characterized by high pOsitivity as well as low Adversity and Duty. Of note, all sport situations scored relatively highly in pOsitivity, reflecting the generally joyful character of sports.

## Discussion

Existing approaches to characterize and distinguish sports typically use simple, ambiguous and unidimensional distinctions that neglect psychologically meaningful features of sports. In this paper, we developed and evaluated a novel way of distinguishing and characterizing sports based on situational characteristics as defined within the DIAMONDS framework [28]. Using ratings by over 7,000 athletes, we derived profiles of more than 100 different sports. These profiles were overall plausible and face valid. For example, team sports like soccer and volleyball generally scored higher on Sociality than individually performed sports like health club training. Mind sports like chess scored higher on Intellect but lower on Sociality and Mating, whereas partner dance scores higher on Sociality, Mating, and pOsitivity, reflecting its joyful and social character. Hence, sports can be meaningfully distinguished and characterized based on their typical situation characteristics.

A unique advantage of our novel approach is that sports are distinguished and characterized more precisely than in previous approaches. For example, team sports are not the only sports that score highly on social situation characteristics such as Sociality and Mating. Partner dance, which is normally not classified as a team sport, has a higher Sociality score than handball, which is typically classified as a team sport. Mating involves the contact to potential sexual and romantic partners and can be a relevant situation characteristic not only in team sports, but also in individual sports such as bodybuilding, which is generally not regarded as a sport involving a relevant amount of social interaction. Hence, individual sports cannot be equated with low or no social interaction, as it has been argued in many studies [13,18].

Additionally, we found that situation characteristics can be used to derive meaningful clusters of sports. We identified seven clusters: *Running & Body Training*, *Aesthetics & Dance Sports*, *Fighting Sports*, *Outdoor Sports*, *Team Sports*, *Mixed*, and *Fun & Dance Sports*. Some of these clusters resemble those of already existing approaches to distinguish sports, such as team versus individual sports [6]. Yet, this cluster also contained sports such as auto racing or eSports, which would not typically be classified as team sports or as sports at all [46]. Further, typically combined sports, such as dance sports, were distributed across two clusters (*Aesthetics & Dance Sports* and *Fun & Dance Sport*s). In conclusion, existing studies that group team sports like basketball, lacrosse, rugby, or soccer together [11] neglect that these sports differ in the way they are perceived as being social as well as in other situation characteristics (see Table 3).

Overall, our results show that we miss relevant psychologically characteristics when grouping sports (e.g. team sports) based on assigned characteristics. Using our approach of including one or several DIAMONDS dimensions in describing sports and physical activity more generally will allow us to investigate underlying mechanisms and relations to variables of interest (e.g. personality) more precisely. If differences between sports on specific psychological variables are of interest, it may be sufficient to concentrate on one or a few of the DIAMONDS dimensions. For example, when investigating the relationship between sports and well-being, the dimension pOsitivity might be most relevant to explain why some sports are associated with higher well-being than others. For other research objectives, however, it might be more appropriate to include all eight DIAMONDS dimensions. For example, the reciprocal relation between sports and personality traits might be mediated by multiple situational characteristics of sports. Additionally, it might make sense to control the situational characteristics of a given sport by collecting these data along with other variables of interest to control for influential factors such as Sociality.

Our study also provides more evidence on the broad applicability and generalizability of the DIAMONDS framework. In past research, the DIAMONDS have been used successfully to describe situations in the context of real-time showed emotions and behavior [29], personnel selection and development, or reciprocal relation of situations and personality [30–33]. Our study showed that the DIAMONDS can also be applied in the context of sports.

### Limitations and future directions

There are some factors limiting the results of the present study. First, for some sports, the consensus at the item level for individual sports was rather low. Thus, the results should be interpreted having that in mind. However, the consensus for items aggregated across sports and the overall agreement for the 24 items of raters for each sport were good. Second, due to the large and growing number of sports performed in the world [1], we were not able to include all sports. However, the sports included in this study were quite representative of the most popular sports given a valid proof of the applicability of the DIAMONDS framework in the context of sports. Moreover, we included the most commonly performed sports and also tried to cover a wide range of different types of sports. Third, we acknowledge that measuring only typical situation characteristics within one sport might neglect the heterogeneity of situations within one sport (e.g., attack versus defense in soccer; regular training vs. competition). This should be considered in future research by examining different situations within one sport. Further, it is possible that some of the variance among athletes within sports is due to systematic individual differences among these athletes in construing the psychological sport situations, selecting certain situations and characteristics within the sport (e.g., being an offense instead of defense player), or both. Future research should examine to what extent the perception of sport situations varies as a function of individual characteristics of athletes, such as their positions, gender, expertise, or personality [47]. In addition, online surveys have the disadvantage that participants can be dishonest or inattentive. We addressed these problems by only including participants who answered all items of the S8*. Further, our participants only received feedback on their sport-specific profile and did not receive any financial compensation. Therefore, we assume our participants to be highly motivated athletes reporting about their sport. Lastly, the calculated consensus measures on the item as well as on the scale level are overall good. Finally, our study provided initial evidence that the DIAMONDS could be applied to sports, but it was beyond the scope of the present study to examine to what extent situation characteristics of sports explain individual differences in psychological correlates of sport and physical activity such as loneliness or well-being over and above established predictors.

## Conclusion

The present study showed that sports can be described and distinguished by their situational characteristics. For single sports, the situational profiles were generally interpretable and face valid. Furthermore, sports could be clustered in meaningful ways into seven classes, each with a distinct DIAMONDS profile. Together, the DIAMONDS framework offered a novel and useful way to distinguish sports in a more comprehensive and psychologically meaningful way, offering a promising pathway towards a better understanding of the relation between sports and psychological variables.

## Supporting information

S1 Table Rank-ordering of the average consensus (ICC) for all sports along with the number (k) of raters after exclusion. Sports with an ICC < .75 (across all S8* items) were excluded (see column “Included”) and sports with k of raters < 2 are not stated.
(PDF)

S2 Table List of sports searched for in the recruiting process or recoded after the data collection. (PDF)

S3 Table Mean values (M) and Standard Deviations (SD) for each of the eight DIAMONDS scales for each sport. (PDF)

S4 Table Cluster numbers for the sports for both types of cluster analyses: Hierarchical and K-means-cluster analysis. (PDF)

S1 Fig Graphical presentation of the mean values (M) and standard errors (SD) for each of the eight DIAMONDS scales for each sport. Sports are sorted alphabetically. The midpoint of the webs reflects a mean value of 1 and the outer line a value of 7. The values for the situational characteristics of the sports are depicted in black. The values for the mean sport situations for the S8* are depicted in green. Standard errors are depicted in dotted lines.
(PDF)

S2 Fig Result of the hierarchical agglomerative cluster analysis for the overall similarity comparison. Clusters framed in red depict clusters with p-values > .95 for 1000 bootstrap repetitions.
(PDF)
