# Supplementary material for: Using the situational characteristics of the DIAMONDS taxonomy to distinguish sports to more precisely investigate their relation with psychologically relevant variables
Source: PLoS One. 2020 Oct 22;15(10):e0241013. doi: 10.1371/journal.pone.0241013 (PMC7581009; doi:10.1371/journal.pone.0241013)
Supplement: S1 Table — Sports with an ICC < .75 (across all S8* items) were excluded (see column “Included”) and sports with k of raters < 2 are not stated. (PDF) [file pone.0241013.s001.pdf]

S1 Table. Rank-ordering of the average consensus (ICC) for all sports along with the number (k) of raters after exclusion. Sports with an ICC < .75 (across all S8* items) were excluded (see column "Included") and sports with k of raters < 2 are not stated.

Sport	ICC(3,*k*)	*k*	Included
Bouldering	.996	249	Yes
Climbing (outdoor)	.996	224	Yes
Ultimate	.996	217	Yes
Rowing	.995	261	Yes
Archery	.994	263	Yes
Partner dance	.994	212	Yes
Ice hockey	.993	164	Yes
Roller derby	.993	115	Yes
eSports	.992	131	Yes
Equestrian vaulting	.991	73	Yes
Horseback riding	.991	152	Yes
Running	.991	227	Yes
(Half-) Marathon	.990	189	Yes
Brazilian jiu-jitsu	.990	115	Yes
Handball	.990	124	Yes
Disc golf	.989	102	Yes
Quidditch	.989	88	Yes
Volleyball	.989	118	Yes
Indoor climbing	.988	86	Yes
Jugger	.988	112	Yes
Paintball & Airsoft	.988	80	Yes
Rugby	.988	104	Yes
Underwater diving	.987	82	Yes
Baseball & Softball	.986	71	Yes
Chess	.986	66	Yes

Cross-country cycling & Mountain biking	.986	118	Yes
Historical European martial arts	.986	83	Yes
Parkour	.986	64	Yes
Mountaineering & Hiking	.985	94	Yes
Pole dance	.985	69	Yes
Shooting sport	.985	70	Yes
Weightlifting	.985	142	Yes
Sailing	.984	65	Yes
Belly dance	.981	53	Yes
Karate	.981	98	Yes
Road bicycle racing	.981	132	Yes
Skydiving	.981	51	Yes
Badminton	.989	83	Yes
CrossFit	.989	66	Yes
Ballet	.979	80	Yes
Paragliding & Hang gliding	.979	54	Yes
Skateboarding	.979	58	Yes
Soccer	.979	79	Yes
Basketball	.978	52	Yes
Table tennis	.977	62	Yes
Trailrunning	.977	76	Yes
Auto racing	.976	41	Yes
Beach volleyball	.975	59	Yes
Surfing	.974	70	Yes
Golf	.973	43	Yes
Jiujitsu	.973	44	Yes
Freediving	.972	56	Yes
Swordsmanship	.972	58	Yes
Aikido	.971	48	Yes
Lacrosse	.971	45	Yes

Taekwondo	.971	53	Yes
Health club training	.970	70	Yes
Snowboarding & Sandboarding	.970	36	Yes
Alpine skiing	.969	45	Yes
Kickboxing	.969	84	Yes
Curling	.968	19	Yes
Obstacle racing	.967	34	Yes
Squash & Racquetball	.967	56	Yes
Windsurfing	.966	35	Yes
Kiteboarding	.965	38	Yes
Longboarding	.965	43	Yes
Triathlon	.965	61	Yes
Calisthenics	.964	42	Yes
Fencing	.964	54	Yes
Capoeira	.963	24	Yes
Tai chi	.962	27	Yes
Underwater rugby	.962	49	Yes
Bodybuilding	.960	60	Yes
Boxing	.959	57	Yes
Bodyweight exercises	.958	61	Yes
Yoga	.958	43	Yes
Field hockey	.957	27	Yes
Gridiron football (including American football)	.957	22	Yes
Judo	.956	39	Yes
Slacklining	.956	32	Yes
Tennis	.954	49	Yes
Wrestling	.954	30	Yes
Floorball	.953	29	Yes
Rafting & Kayaking	.953	18	Yes
Dancing	.952	35	Yes

Sport fishing	.952	26	Yes
Water polo	.951	46	Yes
Bowling	.950	30	Yes
Cricket	.949	20	Yes
Darts	.949	25	Yes
Kendo	.949	35	Yes
Motocross	.946	27	Yes
Boules	.943	18	Yes
Underwater hockey	.941	22	Yes
Canyoning	.938	9	Yes
Flag football	.938	17	Yes
Cross-country skiing	.937	18	Yes
Air sports (planes)	.933	9	Yes
Zumba	.933	19	Yes
Indoor cycling	.929	20	Yes
Table football	.929	20	Yes
Figure skating	.923	21	Yes
Dodgeball & Prisonball	.920	15	Yes
Dragon boat	.918	16	Yes
Synchronized swimming	.914	10	Yes
Australian football & Gaelic football (International rules football)	.912	14	Yes
Mixed martial arts	.907	17	Yes
Canoe polo	.906	12	Yes
Track and field (combined)	.906	18	Yes
Track and field (throwing)	.906	18	Yes
Artistic gymnastics	.903	21	Yes
Tae Bo	.898	11	Yes
Hurling & Shinty	.895	11	Yes
Swimming	.895	31	Yes

Sprinting	.891	24	Yes
Recreational cycling	.888	17	Yes
Krav Maga	.874	19	Yes
BMX	.873	12	Yes
Inline skating	.870	11	Yes
Polo	.869	6	Yes
Trampolining	.864	9	Yes
Breakdancing	.857	13	Yes
Track cycling	.856	13	Yes
Cue sports	.854	8	Yes
Fighting sport - Grappling (Other)	.852	13	Yes
Pole vault	.847	8	Yes
Touch & Tag rugby	.844	7	Yes
Canoeing	.832	12	Yes
Contemporary dance	.828	12	Yes
Racewalking	.826	8	Yes
Aerobics	.819	9	Yes
Cheerleading	.817	6	Yes
Air hockey	.811	4	Yes
Kung fu	.796	10	Yes
Pilates	.795	7	Yes
Qigong	.793	6	Yes
Water skiing	.770	9	Yes
Indoor soccer	.765	6	Yes
Laser tag	.747	10	No
Bobsleigh & Sledding	.741	6	No
Racket sports played with hand	.737	3	No
Speed skating	.693	9	No
Canoe sprint	.674	6	No
Croquet & Gateball	.643	2	No

Gardetanz	.598	2	No
Beach handball	.585	2	No
Nordic walking	.406	3	No
Power walking	.263	2	No
Power boating	.262	2	No
