# Supplementary material for: Using the situational characteristics of the DIAMONDS taxonomy to distinguish sports to more precisely investigate their relation with psychologically relevant variables
Source: PLoS One. 2020 Oct 22;15(10):e0241013. doi: 10.1371/journal.pone.0241013 (PMC7581009; doi:10.1371/journal.pone.0241013)
Supplement: S2 Table — (PDF) [file pone.0241013.s002.pdf]

S2 Table. List of sports searched for in the recruiting process or recoded after the data collection.

Sport category	Sports included in category	Notes
(Half-) Marathon	marathon, half marathon, long-distance running, ultrarunning, endurance running	Recoded: former categories “Long-distance running”, “Jogging” and “Cross-country running”
Aerobics	aerobics, sport aerobics, competitive aerobics	
Aikido	aikido, tomiki aikido, yoshinkan aikido, akikai style, aikido martial art, shodokan	
Air hockey	air hockey	
Air sports (planes)	aerobatics, gliding, air race	
Alpine skiing	alpine skiing, downhill skiing, super giant slalom, super-g, freestyle-skiing, carving, grass skiing, slalom, off-piste, backcountry skiing, freeriding, ski racing, freestyle skiing, ski touring	
Archery	archery, kyūdō, target archery, barebow, compound, recurve archery, traditional archery, olympic archery, warbow archery, longbow archery, primitive archery, traditional asiatic archery, freestyle recurve, 3d archery	
Artistic gymnastics	artistic gymnastics, gymnastics, acro (acrobatic) gymnastics, rhythmic gymnastics, aerial circus arts, floor gymnastics	Recoded: former categories “Rhythmic gymnastics” and “Floor gymnastics” added

Australian football & Gaelic football (International rules football)	australian football, australian rules football, aussie rules, gaelic football, international rules football, german football
Auto racing	formula racing, touring car racing, sports car racing, stock car racing, rallying (road vs stage), drag racing, off-road racing, kart racing, endurance racing, drifting, drag racing, time attack, midget, speedcar racing, production car racing
Badminton	badminton, ball badminton, beachminton, speedminton, crossminton, (battledore and shuttlecock)
Ballet	ballet, classical ballet, neoclassical, vaganova method, pointe, non-pointe
Bandy	Bandy
Baseball & Softball	baseball, softball, pesäpollo, finish baseball, lapta, palant, oina, kinball, langbold, longaméta, fastball
Basketball	basketball, netball, streetball, street basketball, slamball
Baton twirling	baton twirling
Beach handball	beach handball
Beach volleyball	beach volleyball
Beach basketball	beach basketball
Belly dance	belly dance, tribal style (american/egyptian), tribal fusion, cabaret style
Biathlon	Biathlon
BMX	BMX, bicycle motocross, freestyle BMX, street BMX, BMX racing, dirt BMX

Bobsleigh & Sledding	bobsleigh, bobsled, skeleton, luge, sledding, sledging	
Bodybuilding	bodybuilding, classic physique, powerbuilding, aesthetics	
Bodyweight exercises	bodyweight exercises, Freeletics, convict conditioning	
Bouldering	bouldering, indoor, outdoor, competitive, gym bouldering	
Boules	bocce, pétanque, bocce volo, boccia, jeu provencal, boule bretonne, boule-des-berges, boule-de-fort, boule-en-bois, petanque	
Bowling	ten-pin bowling, bowling, 9-pin bowling	
Boxing	boxing, pugilism, amateur boxing	
Brazilian Jiu Jitsu	brazilian jiu-jitsu	Added: former categories “Fighting sports - grappling” or “Mixed (hybrid) martial arts”
Breakdancing	b-boying, breaking, breakdancing, popping	
Calisthenics	Calisthenics	
Canoe polo	canoe polo	
Canoe sprint	canoe sprint, marathon canoeing, flat water racing	
Canoeing	whitewater canoeing, slalom canoeing, freestyle canoeing, outrigger canoe, long distance canoeing	
Canyoning	Canyoning	
Capoeira	capoeira, contemporary capoeira, capoeira regional	
Cheerleading	Cheerleading	

Chess	chess, over the board chess vs. online chess, speed chess, tournament chess	
Cliff diving	cliff diving	
Climbing (outdoor)	ice climbing, buildering, competition climbing, alpine climbing, free climbing, sport climbing, trad (traditional)climbing, top rope climbing, lead climbing, rock climbing, multi-pitch climbing	
Contemporary dance	jazz dance, contemporary dance, modern dance	
Cricket	cricket, vigoro, cricko	
Croquet & Gateball	croquet, roque, gateball	
Cross-country cycling & Mountain biking	mountain bike racing, cross-country cycling, mtb, cyclo-cross, cyclocross, cx, 'cross, cyclo-x, enduro, freeride, gravel riding, downhill	
Cross-country skiing	cross-country skiing, classic style, skate style, back country skiing	
CrossFit	CrossFit	
Cue sports	russian pyramid, russian billiard, pyramid billiards, pool billiards, pocket billiards, snooker, cue sports, cue sport, billiard sports, english billiards, 8ball	
Curling	curling, ice stock sport, bavarian curling	
Dancing	eurodance, dancefloor, disco dance, hip-hop, shuffling, popping, tap dancing, irish dance, tap dance	Recoded: former category “Tap dance” added
Darts	darts, steeldarts	
Dragon boat	dragon boat	Added: former categories “Rowing” or “Canoeing”
Disc golf	disc golf	

High Diving	diving, competitive diving, high diving, artistic diving
Dodgeball & Prisonball	dodgeball, prisonball, nationball, battleball, trench, jail ball, jail dodgeball, greek dodgeball, german dodgeball, teamball, crossfire, warball, swedish dodgeball, dungeon dodge, king's court in Canada, heaven in New Zealand
Equestrian vaulting	equestrian vaulting, vaulting
eSports	eSports, electronic sports, competitive (video) gaming, professional (video) gaming, or pro gaming
Fencing	olympic fencing, fencing, sabre, foil, epee, rapier
Field hockey	field hockey, street hockey, deck hockey, ball hockey, road hockey
Fighting sport - Grappling (Other)	luta livre, nihon jujutsu, judo, shuaijiao, quin na, kenpo, etc.
Figure skating	figure skating (single, pair skating, ice dancing), synchronized skating, show skating
Fistball	Fistball
Flag Football	flag football, touch football, american flag football
Floorball	floorball, floor hockey
Footgolf	Footgolf
Freediving	freediving, free-diving, free diving, breath-hold diving, skin diving, recreational freediving
Gardetanz	Gardetanz, show dance
Golf	golf, urban golf, swingolf, crossgolf

Gridiron football (including American football)	arena football, indoor gridiron football, american football, north american football, american football, canadian football	
Handball	field handball, outdoor handball, grass handball, team handball, olympic handball	
Health club training	health club, fitness club, fitness center, health spa, gym	
High jump	high jump	
Historical European Martial Arts	historical european martial arts, HEMA, historical fencing	Added: former categories “Swordsmanship” and “Fencing”
Horseback riding	horseback riding, riding, equestrianism, "western" riding, horse racing, dressage, show jumping, stadium jumping, open jumping	
Hurling & Shinty	hurling, camogie, shinty	
Ice hockey	ice hockey, ringette, recreational ice hockey	
Indoor climbing	indoor climbing, top roping, lead climbing, sport climbing	
Indoor cycling	indoor cycling, spinning	
Indoor Soccer	indoor soccer, futsal	
Inline skating	inline skating, rollerblading, skating, inline speedskating, freestyle skating, distance skating, freestyle slalom	
Jiu Jitsu	jiujitsu (submission grappling, traditional, japanese, martial arts)	
Judo	Judo	
Jugger	Jugger	
Karate	karate (different types)	

Kendo	kendo, japanese fencing	Added: former categories “Swordsmanship” and “Fencing”
Kickboxing	savate, boxe française, french boxing, french kickboxing, french footfighting, muay thai	
Kiteboarding	kitesurfing, kiteboarding, snowkiting, kitesailing, kite bugging, freeride kiteboarding, kitefoiling	
Krav Maga	krav maga	Added: former category “Fighting sports – grappling”
Kung fu	kung fu, wing chun, ving tsun	Added: former category “Fighting sports – grappling”
Lacrosse	lacrosse, intercrosse, box lacrosse, indoor lacrosse, box, boxla, field lacrosse	
Laser tag	laser tag	
Long jump	long jump, triple jump	
Longboarding	longboarding, longboard dancing, freeride, downhill, long distance pumping, cruising, freestyle	
Miniature golf	mini-golf, putt-putt, adventure golf	
Mixed Martial Arts	mixed martial arts	
Motocross	supercross, quad motocross, speedway, freestyle-motocross, ice speedway, motoball, track racing, enduro, dirt bike	
Mountaineering & Hiking	mountaineering, mountain climbing, ski mountaineering, hiking in the mountains, alpinism, hiking, backpacking, peak climbing/hiking, scrambling	

Nordic combined	nordic combined (country skiing & ski jumping)	
Nordic Walking & Walking	nordic walking, power nordic walking, walking	Recoded: former category “Power walking” added
Obstacle racing	obstacle racing, rugged maniac, spartan race, tough mudder, etc.	
Paintball & Airsoft	paintball, airsoft, softair, skirmish, woodsball/ rec ball, milsim, speedball paintball,	
Paragliding & Hang gliding	paragliding, paramotor, free flight, acro paragliding, cross country paragliding, hang gliding	
Parkour	Parkour	
Partner dance	swing dance, latin, rhythm, salsa, rock'n'roll, ballroom dancing, lindy hop, blues, tango	
Pilates	pilates, reforming, reformer	
Pole dance	pole dance, pole fit/fitness, pole sport, contemporary pole	
Pole vault	pole vault	
Polo	polo, pato, cycle polo, bike polo, polocrosse, elephant polo	
Power boating	power boating, yachting, jet skiing	
Qigong	qigong, qi gong, chi kung, chi gung	
Quidditch	Quidditch	
Racewalking	race-walking, race walking	
Racket sports played with hand	court handball, handball, american handball, welsh handball, gaelic handball, australian handball, fives, eton fives, city fives, rugby fives, irish handball, pelato, jai alai, cesta-punta	

Rafting & Kayaking	rafting, white water rafting, kayaking	Recoded: “kayaking” was added
Recreational cycling	cycling, road cycling (not racing), bike riding, cycling	Added: former categories “Cross-country cycling” and “Road bicycle racing”
Road bicycle racing	road bicycle racing, crit (criterium) racing, alley cat racing, randonneuring, road cycling, gravel	
Roller derby	roller derby, merby, flat track	
Rope Skipping	rope skipping, jump rope	
Rowing	rowing, sweep rowing/sweeping, sculling, erging (ergometer)	
Rugby	rugby, rugby league, rugby union, rugby sevens, rugby tens, ten-a-side, seven-a-side	
Running	short distance, medium distance, jogging	Recoded: former categories “Jogging”, “Long-distance running”, “Cross country running”
Sailing	sailing, racing, cruising, yacht racing	
Shooting sport	shooting sport, plinking, sporting clays, clay pigeon shooting, golf with a shotgun, trap shooting, pistol shooting, speed shooting, action shooting	
Skateboarding	skateboarding, street, park, ramp, halfpipe, bowl, cruising	
Ski jumping	ski jumping, sky flying	
Skydiving	skydiving, parachuting, bungee-jumping, base-jumping, base jumping, wingsuit skydiving, swooping, formation skydiving, free fly	

Slacklining	slacklining, highlining, longlining, tricklining	
Snowboarding & Sandboarding	snowboarding, sandboarding, skwal	
Soccer	association football, soccer, beachsoccer	
Speed skating	speed skating, long-track speed skating, short track, short track skating, marathon speed skating	
Sport fishing	angling, fishing, sport fishing, recreational fishing, fly fishing, bass fishing, offshore fishing	
Sprinting	relay race, relay, sprinting, 50 to 800m, hurdling	Recoded: former category "Hurdling" added
Squash & Racquetball	racquetball, squash 57, racketball, rackets, ricochet	
Surfing	surfing, bodyboarding, stand up paddle surfing, stand up paddle boarding, sup, bodysurfing, bodyboarding, skimboarding	
Swimming	swimming, open water swimming, finswimmimg	
Swordsmanship	swordfighting, swordsmanship, naginata, jogo do pau, haidong gumdo, haedong kumdo, hangeomdo, iaidō, kenjutsu, kumdo, gumdo, german longsword, longsword fencing, iaido	
Synchronized diving	synchronized diving	
Synchronized swimming	synchronized swimming, water ballet	
Table football	table football, fuzboll, foosball, table soccer	
Table tennis	table tennis, ping pong	
Tae Bo	tae bo	

Taekwondo	Taekwondo	
Tai chi	tai chi (different types)	
Tennis	Tennis	
Touch & Tag rugby	touch rugby, tag rugby	
Track and field (combined)	track and field (combined events), men's decathlon, women's heptathlon, men's heptathlon (indoor), women's pentathlon (indoor), track and field, track and field (running events - short and middle distance)	
Track and field (throwing)	javelin throw, shot put, discus throw, hammer throw	
Track cycling	track cycling, endurance track cycling, sprint	
Trailrunning	trailrunning, mountain running, cross country running, fell running, hill running	Recoded: former categories “Cross country running”
Trampolining	trampolining, synchronized trampoline, tumbling, power tumbling	
Triathlon	triathlon, ironman, duathlon	
Truck and Tractor pulling	truck and tractor pulling, power pulling	
Ultimate	ultimate, ultimate frisbee	
Underwater Diving	underwater diving, underwater orienteering, scuba diving, cave diving	
Underwater hockey	underwater hockey, octopush	
Underwater rugby	underwater rugby	
Volleyball	Volleyball	
Water aerobics	water aerobics, waterobics, aquatic fitness, aqua fitness, aquafit	
Water polo	water polo	
Water skiing	waterskiing, wakeboarding, kneeboarding	

Weightlifting	weightlifting, olympic-style weightlifting, olympic weightlifting, powerlifting, strongman
Wheel gymnastics	wheel gymnastics, gym wheel, german wheel, ayro wheel, rhon rod, aero wheel
Windsurfing	windsurfing, wave sailing, freestyle, freeride
Wrestling	greco-roman wrestling, freestyle wrestling, female wrestling, oil wrestling, sumo, koshti, boke, sambo, freestyle wrestling, folkstyle
Yoga	yoga, aerial yoga, bikram-yoga, hormonyoga, poweryoga, vinyasa, ashtanga, power yoga, hatha yoga
Zumba	Zumba
