# Supplementary material for: Using the situational characteristics of the DIAMONDS taxonomy to distinguish sports to more precisely investigate their relation with psychologically relevant variables
Source: PLoS One. 2020 Oct 22;15(10):e0241013. doi: 10.1371/journal.pone.0241013 (PMC7581009; doi:10.1371/journal.pone.0241013)
Supplement: S3 Table — (PDF) [file pone.0241013.s003.pdf]

S3 Table. Mean values (M) and Standard Deviations (SD) for each of the eight DIAMONDS scales for each Sport.

	Duty		Intellect		Adversity		Mating		pOsitivity		Negativity		Deception		Sociality	
Sport	*M*	*SD*	*M*	*SD*	*M*	*SD*	*M*	*SD*	*M*	*SD*	*M*	*SD*	*M*	*SD*	*M*	*SD*
(Half-) Marathon	4.01	1.67	2.60	1.40	1.56	0.81	2.25	1.14	4.39	1.16	3.76	1.61	2.23	1.22	3.32	1.49
Aerobics	4.33	2.19	3.70	2.10	2.67	1.14	2.74	1.44	5.33	1.21	4.04	1.60	2.30	1.09	5.00	1.01
Aikido	4.27	1.35	5.06	1.22	2.90	1.33	1.91	0.89	5.33	1.10	4.51	1.62	3.01	1.73	5.08	1.09
Air hockey	5.50	1.29	5.75	1.17	2.08	1.13	2.00	1.05	5.42	0.74	5.58	1.29	4.00	1.19	5.25	1.85
Air sports (planes)	6.07	0.72	5.67	1.30	2.30	0.89	1.44	0.67	4.44	1.17	5.70	1.22	2.26	1.87	4.48	1.67
Alpine skiing	4.41	1.82	3.99	1.63	1.82	0.88	2.59	1.40	5.83	1.29	4.74	1.63	2.35	1.31	4.36	1.56
Archery	4.12	1.52	3.92	1.47	1.76	0.81	2.03	1.06	4.80	1.13	4.06	1.68	2.18	1.21	4.07	1.47
Artistic gymnastics	4.54	1.57	3.78	1.68	2.63	1.40	3.06	1.32	5.06	1.34	4.92	1.44	2.49	1.80	4.70	1.63
Australian football & Gaelic football (International rules football)	5.98	0.83	4.69	1.47	3.62	1.81	1.74	1.04	5.07	1.79	5.50	0.84	3.79	1.66	6.05	0.68
Auto racing	6.05	1.05	5.54	1.38	3.17	1.35	1.73	0.88	4.88	1.32	6.37	0.78	4.56	1.97	5.14	1.46

Badminton	4.59	1.40	4.27	1.55	2.43	1.07	2.42	0.99	5.27	1.18	4.89	1.20	4.54	1.36	4.93	1.28
Ballet	4.88	1.37	5.06	1.42	3.19	1.39	3.73	1.39	4.97	1.10	5.14	1.44	2.85	1.58	4.80	1.31
Baseball & Softball	5.94	1.24	4.86	1.58	2.75	1.29	1.91	0.89	5.08	1.08	5.24	1.35	3.75	1.60	5.86	1.12
Basketball	5.32	1.30	4.38	1.26	3.03	1.33	1.90	0.97	5.26	1.09	4.56	1.32	3.79	1.56	5.83	0.96
Beach volleyball	4.44	1.53	3.62	1.29	2.44	0.92	4.16	1.30	5.66	0.96	4.25	1.45	3.20	1.34	5.71	1.00
Belly dance	4.33	1.54	4.92	1.45	2.16	1.24	3.43	1.44	6.20	0.76	3.69	1.27	2.31	1.45	5.35	1.20
BMX	3.36	1.45	3.50	1.52	1.89	0.82	2.47	1.25	5.78	1.25	4.28	1.95	2.56	1.47	4.08	1.36
Bodybuilding	5.39	1.47	3.27	1.61	2.29	1.12	4.41	1.62	3.84	1.43	3.88	1.74	3.03	1.76	3.32	1.38
Bodyweight exercises	4.30	1.57	2.86	1.60	1.66	0.82	2.80	1.45	4.53	1.29	3.68	1.65	2.39	1.47	3.05	1.66
Bouldering	4.42	1.15	4.38	1.40	1.64	0.73	3.15	1.20	5.69	0.91	4.33	1.42	1.92	1.05	4.99	1.31
Boules	5.33	1.13	3.89	1.32	2.56	1.12	1.80	0.67	5.13	0.96	5.07	1.11	3.57	1.71	5.20	1.16
Bowling	4.61	1.15	4.09	1.46	2.28	1.24	2.01	0.99	4.76	1.48	5.06	1.11	2.40	1.56	4.48	1.28
Boxing	5.22	1.49	4.93	1.60	3.64	1.35	2.18	1.33	4.40	1.50	5.27	1.50	4.12	1.59	4.41	1.51
Brazilian jiu-jitsu	4.75	1.51	6.04	1.08	3.37	1.32	1.88	0.96	5.05	1.19	5.87	1.05	4.08	1.62	4.99	1.26
Breakdancing	3.82	1.83	3.23	2.00	2.87	1.17	3.10	1.43	5.67	1.52	5.44	1.57	3.10	2.00	5.69	1.58
Calisthenics	4.52	1.51	3.48	1.36	1.60	0.81	2.47	1.08	5.32	1.23	3.35	1.42	2.24	1.35	3.37	1.65
Canoe polo	5.44	1.20	3.78	1.21	3.03	1.18	2.47	1.39	5.25	1.20	5.17	1.43	3.61	1.68	5.61	0.75

Canoeing	3.69	1.35	3.69	0.78	2.53	1.59	2.19	1.28	5.28	1.09	4.58	2.18	2.72	1.99	4.53	1.65
Canyoning	6.48	0.50	4.74	0.85	2.52	1.16	2.37	0.99	5.30	1.32	4.74	1.05	1.74	0.92	5.74	1.10
Capoeira	3.75	1.62	5.58	1.22	2.68	0.99	3.31	1.19	6.49	0.71	4.28	1.16	5.81	1.17	6.43	0.60
Cheerleading	5.89	0.75	3.44	1.03	2.67	1.17	3.94	1.24	5.22	0.93	3.50	1.99	2.83	1.13	5.89	1.07
Chess	4.70	1.62	6.64	0.73	2.94	1.53	1.59	1.02	4.43	1.31	5.76	1.23	4.08	1.69	2.88	1.33
Climbing (outdoor)	5.30	1.45	4.96	1.52	2.12	1.10	2.91	1.20	5.37	1.07	5.54	1.35	2.18	1.31	5.73	1.11
Contemporary dance	4.61	1.91	4.31	1.57	3.47	1.42	4.08	1.79	5.56	1.48	4.64	2.00	3.17	1.37	5.92	1.24
Cricket	5.82	0.98	5.47	1.24	3.70	1.51	1.77	1.23	5.12	1.13	5.88	1.47	4.20	1.18	5.85	0.95
Cross-country cycling & Mountain biking	3.62	1.50	3.17	1.31	1.95	1.05	2.37	1.17	5.59	1.08	4.07	1.52	2.32	1.41	4.11	1.51
Cross-country skiing	4.17	1.71	3.44	0.97	1.56	0.74	2.44	1.01	5.48	1.29	4.19	1.50	1.85	0.76	3.72	1.23
CrossFit	4.94	1.30	3.13	1.39	1.68	0.74	3.36	1.30	5.11	1.06	3.86	1.48	2.74	1.32	5.36	1.26
Cue sports	5.50	1.21	6.33	1.01	3.25	1.99	2.92	2.16	5.83	0.73	6.21	0.69	3.54	2.01	4.13	1.93
Curling	5.79	1.25	5.67	1.42	2.72	1.46	1.95	0.82	4.96	0.89	5.37	1.47	2.53	1.32	6.21	1.13
Dancing	4.00	2.05	4.47	1.80	2.63	1.35	4.20	1.84	5.90	0.99	4.68	1.76	2.79	1.39	5.50	1.51
Darts	4.97	1.59	3.59	1.44	2.35	1.30	1.48	0.87	5.33	1.28	5.44	1.25	3.05	1.78	4.11	1.39

Disc golf	4.34	1.61	3.83	1.54	1.60	0.70	1.65	0.75	5.80	1.06	4.72	1.49	2.92	1.55	4.15	1.42
Dodgeball & Prisonball	5.42	1.09	4.53	1.26	3.80	1.07	2.98	0.99	5.80	0.84	5.76	0.84	5.16	1.28	6.33	0.50
Dragon boat	4.40	1.65	3.08	1.22	2.67	1.14	2.42	1.03	4.50	1.12	3.73	1.54	1.79	0.70	5.19	1.39
Equestrian vaulting	5.42	1.07	3.57	1.40	2.36	0.84	2.44	0.81	5.08	1.06	4.30	1.48	1.86	0.92	5.98	1.16
eSports	5.84	1.16	5.41	1.28	4.38	1.30	1.58	0.94	4.88	1.18	5.91	1.06	4.17	1.49	5.60	0.96
Fencing	4.54	1.48	5.38	1.27	3.23	1.31	2.31	0.97	5.30	1.20	5.22	1.35	4.62	1.57	4.52	1.25
Field hockey	5.11	1.26	4.04	1.08	2.64	1.19	2.28	1.06	5.26	1.07	4.44	1.33	3.17	1.28	5.54	1.13
Fighting sport - Grappling (Other)	5.15	1.58	5.23	0.91	3.41	1.33	2.23	1.02	4.38	0.99	5.05	1.62	3.46	1.91	5.13	1.07
Figure skating	5.27	1.57	4.97	1.59	3.27	1.32	3.79	1.42	5.24	1.23	5.70	1.13	3.29	1.58	4.52	1.72
Flag football	5.82	1.24	4.63	1.50	2.92	1.28	1.94	1.05	5.29	1.14	4.51	1.20	4.12	1.44	5.76	0.96
Floorball	5.05	1.23	3.43	1.41	2.24	1.00	2.44	1.36	5.59	1.16	4.28	1.52	3.33	1.44	5.61	1.13
Freediving	4.06	1.58	3.92	1.41	1.83	1.13	2.30	1.06	4.73	1.30	3.97	1.69	1.81	0.94	4.82	1.25
Golf	4.84	1.16	5.14	1.20	2.25	1.05	1.91	0.98	4.95	0.94	5.86	1.28	4.05	1.42	3.97	1.53
Gridiron football (including	6.23	0.76	4.44	1.42	3.92	1.17	1.64	0.77	4.33	0.99	4.95	0.82	3.67	1.41	5.89	1.05

American football)																
Handball	5.59	1.04	3.92	1.56	3.14	1.14	2.55	1.26	4.87	1.03	4.81	1.14	3.40	1.29	5.99	0.79
Health club training	4.21	1.56	2.40	1.38	1.64	0.95	3.62	1.47	3.50	1.04	3.03	1.45	2.36	1.30	3.29	1.46
Historical European martial arts	4.69	1.49	5.82	1.27	3.40	1.15	1.90	0.83	5.52	1.08	5.53	1.21	4.57	1.66	5.22	1.35
Horseback riding	5.66	1.10	4.26	1.55	2.87	1.22	2.21	0.99	5.07	1.01	4.94	1.42	3.29	1.60	4.68	1.40
Hurling & Shinty	5.67	0.91	4.21	1.35	3.36	1.49	2.21	1.07	5.09	0.99	5.03	1.18	3.73	1.50	5.88	0.89
Ice hockey	5.55	1.14	4.07	1.36	3.72	1.36	1.73	1.01	5.29	1.07	5.54	1.16	4.04	1.50	5.87	0.93
Indoor climbing	4.76	1.37	4.37	1.50	1.83	0.78	2.90	1.19	5.34	0.88	4.82	1.38	2.09	1.19	5.28	1.27
Indoor cycling	4.03	1.32	2.40	1.20	1.37	0.68	2.65	1.07	5.03	1.25	2.53	1.46	2.35	1.51	4.13	1.79
Indoor soccer	4.33	1.55	3.28	1.84	3.00	1.85	2.33	1.03	4.89	0.40	4.28	1.29	3.89	0.75	5.22	0.86
Inline skating	2.58	1.54	2.73	1.50	2.06	1.31	2.39	1.52	6.03	0.90	3.55	1.50	1.70	1.16	4.00	2.06
Jiujitsu	4.92	1.44	5.77	1.46	3.02	1.26	2.13	1.23	5.45	1.13	5.67	1.37	4.11	1.61	5.22	1.16
Judo	5.03	1.28	5.06	1.07	2.60	1.08	2.10	1.00	4.64	1.46	4.76	1.69	3.32	1.51	4.90	1.25
Jugger	5.43	1.10	4.22	1.29	2.97	1.27	3.01	1.18	5.70	1.01	4.42	1.17	4.32	1.33	6.15	0.68

Karate	4.76	1.43	4.98	1.42	2.97	1.28	1.98	0.91	4.54	1.11	4.20	1.47	3.03	1.51	4.87	1.35
Kendo	4.80	1.45	5.02	1.28	3.89	1.22	1.96	0.85	4.30	1.36	5.61	1.14	3.97	1.56	4.70	1.34
Kickboxing	4.81	1.47	4.54	1.59	3.23	1.38	2.36	1.11	4.67	1.15	5.27	1.48	4.26	1.66	4.74	1.42
Kiteboarding	3.49	1.69	3.85	1.85	2.26	0.94	2.77	1.35	5.99	0.85	4.60	1.34	2.06	1.07	4.64	1.34
Krav Maga	5.16	1.20	4.32	1.50	3.40	1.43	2.21	0.96	3.81	1.89	5.39	1.33	3.63	2.20	4.88	1.38
Kung fu	4.63	1.19	5.27	1.10	2.60	1.48	1.83	0.74	4.70	1.76	4.20	1.49	3.57	1.45	5.07	1.14
Lacrosse	5.70	1.15	4.58	1.27	3.19	1.31	2.38	1.51	5.36	0.91	4.98	1.38	3.66	1.47	6.21	0.80
Longboarding	2.80	1.39	3.27	1.70	2.26	1.24	2.33	1.26	6.20	0.96	4.36	1.68	2.62	1.43	4.44	1.45
Mixed martial arts	4.82	1.33	5.35	1.21	2.88	1.44	1.80	1.01	4.82	1.23	4.69	1.64	3.02	1.55	5.06	1.36
Motocross	4.32	1.85	4.01	1.63	2.37	1.51	2.05	1.38	5.79	1.07	5.25	1.63	2.94	1.69	3.53	1.45
Mountaineering & Hiking	4.67	1.76	4.36	1.54	1.97	1.10	2.62	1.18	5.12	1.19	4.69	1.75	2.29	1.21	4.83	1.51
Obstacle racing	5.22	1.35	2.71	1.45	1.41	0.65	3.05	1.39	5.37	1.25	4.73	1.45	2.39	1.51	4.31	1.56
Paintball & Airsoft	5.64	1.21	4.41	1.40	3.00	1.19	1.64	0.82	5.59	1.08	5.47	1.21	4.74	1.43	5.87	0.86
Paragliding & Hang gliding	4.49	1.76	4.75	1.48	2.10	1.17	1.90	0.95	5.70	1.04	4.98	1.62	2.27	1.21	4.04	1.37
Parkour	3.95	1.60	4.52	1.49	1.93	1.06	2.15	1.06	6.33	0.72	4.54	1.45	2.08	1.26	5.25	1.22
Partner dance	4.02	1.75	4.41	1.65	2.61	1.24	4.85	1.34	6.15	0.93	4.36	1.60	2.82	1.57	6.24	0.87
Pilates	3.71	1.53	2.48	1.67	1.19	0.33	2.05	0.62	4.67	1.83	3.57	1.67	1.86	1.09	2.86	1.69

Pole dance	3.53	1.48	3.73	1.46	1.63	0.68	3.05	1.50	5.93	0.97	3.51	1.37	1.88	1.03	4.86	1.36
Pole vault	5.33	1.48	5.42	1.11	2.42	0.75	2.33	1.63	5.21	1.33	5.29	2.03	2.83	1.47	4.00	1.39
Polo	6.39	1.18	5.06	1.45	3.17	1.35	2.61	0.93	6.06	0.85	5.44	1.22	3.17	1.33	6.44	0.54
Qigong	3.22	2.08	3.44	1.44	1.56	0.62	1.67	0.84	5.17	1.05	2.28	1.25	2.11	1.77	2.56	1.11
Quidditch	5.33	1.14	4.74	1.13	2.74	1.33	3.13	1.13	5.55	1.01	4.77	1.30	3.59	1.42	6.19	0.80
Racewalking	5.17	1.55	3.75	1.44	2.29	1.03	1.58	0.56	4.46	1.47	4.88	1.95	3.04	1.69	3.96	1.79
Rafting & Kayaking	5.37	1.21	4.85	1.48	2.41	1.27	2.30	1.11	5.65	1.25	5.50	1.42	2.17	1.67	5.78	1.01
Recreational cycling	3.55	1.56	3.29	1.88	1.92	1.07	2.25	1.59	5.41	1.10	4.33	1.70	2.78	1.94	4.43	1.71
Road bicycle racing	4.28	1.74	3.49	1.62	2.45	1.33	2.23	1.21	4.55	1.27	4.70	1.60	4.10	1.77	4.45	1.54
Roller derby	5.59	1.07	4.81	1.25	2.64	1.10	2.47	1.07	5.59	0.96	5.55	1.14	3.61	1.36	6.39	0.65
Rowing	5.84	1.00	3.75	1.45	3.30	1.32	2.75	1.30	3.89	1.38	5.50	1.30	2.47	1.29	5.42	1.20
Rugby	5.79	1.02	4.18	1.33	3.07	1.19	2.56	1.19	5.14	1.01	4.71	1.35	3.26	1.32	6.27	0.77
Running	3.67	1.67	2.44	1.38	1.49	0.78	2.29	1.16	4.35	1.26	3.46	1.57	1.98	1.15	3.06	1.54
Sailing	5.77	1.35	5.02	1.40	2.56	1.38	2.44	1.19	4.81	1.14	5.01	1.37	2.40	1.33	5.10	1.48
Shooting sport	5.38	1.30	4.74	1.41	2.20	1.23	1.68	0.96	4.95	1.25	5.39	1.40	2.30	1.42	4.24	1.54
Skateboarding	2.95	1.40	3.47	1.81	2.06	1.20	2.07	1.08	6.00	1.03	4.76	1.59	2.03	1.20	4.02	1.49
Skydiving	5.60	1.22	4.74	1.41	2.73	1.21	2.95	1.28	6.07	0.85	5.41	1.23	2.58	1.48	5.82	1.11
Slacklining	3.49	1.43	3.52	1.44	1.78	0.86	2.67	1.28	5.79	1.12	4.24	1.86	1.71	1.13	4.36	1.36

Snowboarding & Sandboarding	3.14	1.60	3.46	1.39	1.83	1.00	2.44	1.17	6.15	0.89	4.29	1.56	2.12	1.08	4.60	1.39
Soccer	5.16	1.55	3.89	1.44	3.27	1.49	2.16	1.16	5.10	1.13	4.75	1.39	3.62	1.59	5.65	1.00
Sport fishing	4.18	1.39	4.47	1.59	1.86	0.85	1.49	0.90	5.38	1.11	4.79	1.60	3.04	1.68	3.67	1.50
Sprinting	5.15	1.55	3.49	1.59	2.54	1.47	3.49	1.72	4.69	1.18	4.72	1.86	3.11	1.68	4.58	1.61
Squash & Racquetball	3.89	1.75	4.06	1.42	2.04	1.20	1.98	1.32	5.23	1.02	4.80	1.34	4.25	1.58	3.96	1.35
Surfing	2.82	1.31	3.46	1.51	2.86	1.51	2.83	1.31	6.21	0.89	4.72	1.54	3.19	1.71	3.95	1.20
Swimming	4.41	1.69	3.55	1.60	2.22	1.32	3.28	1.57	4.62	1.38	3.97	1.70	2.60	1.26	4.15	1.55
Swordsmanship	4.58	1.49	5.79	0.88	3.23	1.23	2.16	0.93	5.23	1.20	5.10	1.51	4.14	1.83	5.25	1.26
Synchronized swimming	5.77	1.12	5.07	1.79	2.97	1.47	2.60	0.83	5.77	1.03	4.90	1.64	2.13	1.57	5.97	0.95
Table football	5.02	1.48	4.70	1.49	2.48	0.93	2.30	1.01	4.88	0.90	5.90	0.86	4.28	1.42	4.70	1.28
Table tennis	4.59	1.24	4.82	1.54	2.17	0.95	1.74	0.80	4.95	1.16	4.99	1.30	4.02	1.60	4.50	1.23
Tae Bo	3.82	1.50	3.18	1.32	1.76	0.52	2.42	0.54	5.15	1.13	2.76	1.38	1.45	0.45	4.55	1.45
Taekwondo	5.16	1.58	4.92	1.49	2.93	1.41	2.13	1.06	5.04	1.09	4.74	1.37	3.04	1.52	5.24	1.14
Tai chi	4.00	1.83	5.70	1.32	1.78	0.84	1.79	1.21	5.17	1.09	2.93	1.28	2.10	1.07	4.05	1.63
Tennis	4.50	1.30	4.41	1.93	2.54	1.25	2.24	1.26	4.57	1.24	5.45	1.44	4.43	1.75	4.31	1.54
Touch & Tag rugby	5.10	1.33	3.90	1.57	2.71	1.08	3.29	1.08	6.14	0.74	4.48	1.51	3.95	1.46	6.48	0.54

Track and field (combined)	5.33	1.02	4.07	1.48	2.56	0.97	3.04	1.27	4.56	1.33	5.37	1.20	3.22	1.60	5.00	1.09
Track and field (throwing)	4.35	1.57	4.09	1.77	2.65	1.36	2.07	1.02	4.54	1.44	4.74	1.55	1.94	0.94	4.20	1.61
Track cycling	4.69	1.21	4.44	1.29	2.38	0.84	2.49	1.56	4.49	1.37	5.54	0.89	4.08	1.64	5.13	1.27
Trailrunning	3.89	1.76	3.03	1.49	1.49	0.79	2.33	1.11	4.73	1.21	3.98	1.47	2.12	1.16	3.62	1.51
Trampolining	4.81	1.80	4.96	1.66	2.22	0.94	3.15	1.71	5.41	1.66	4.81	1.37	2.41	0.64	4.81	1.32
Triathlon	4.64	1.33	2.83	1.48	1.70	0.81	2.80	1.28	4.04	1.27	4.04	1.38	2.74	1.30	3.47	1.54
Ultimate	4.92	1.12	4.44	1.26	2.36	0.94	2.89	0.98	5.67	0.84	4.42	1.35	3.51	1.44	6.06	0.84
Underwater diving	4.89	1.55	4.54	1.43	2.11	1.10	2.25	0.99	5.13	1.12	4.48	1.36	2.41	1.23	5.41	1.27
Underwater hockey	5.65	0.81	4.30	1.55	2.79	1.05	2.68	0.71	5.39	1.01	4.58	1.20	3.36	1.54	5.17	1.10
Underwater rugby	5.37	1.26	3.88	1.48	3.08	1.28	2.69	1.02	5.13	1.14	4.84	1.28	3.29	1.30	5.04	1.22
Volleyball	5.58	1.13	4.21	1.52	3.01	1.42	2.61	1.17	5.44	0.96	4.90	1.25	3.96	1.56	6.16	0.72
Water polo	5.44	1.04	3.77	1.41	3.62	1.36	2.68	1.38	4.74	1.21	5.03	1.20	3.94	1.47	5.66	1.15
Water skiing	4.15	1.30	3.56	1.00	2.67	1.40	3.37	1.81	5.44	1.08	4.26	1.27	2.59	1.26	4.52	1.26
Weightlifting	5.08	1.48	3.56	1.67	2.18	1.06	2.71	1.47	4.35	1.35	4.83	1.50	2.65	1.61	3.59	1.58
Windsurfing	2.92	1.53	3.11	1.34	1.78	1.06	2.90	1.32	6.02	0.94	3.91	1.77	1.78	1.02	4.35	1.61
Wrestling	5.36	1.58	4.70	1.39	4.16	1.47	1.57	0.86	3.58	1.28	6.12	0.94	4.40	1.70	4.24	1.42

Yoga	2.68	1.63	3.47	1.63	1.28	0.57	2.17	1.05	4.79	1.12	2.60	1.20	1.89	0.93	2.98	1.62
Zumba	2.96	1.76	2.96	1.79	1.65	1.13	2.84	1.38	5.98	0.89	2.40	1.60	1.84	1.40	3.58	1.76
