# Supplementary material for: Using the situational characteristics of the DIAMONDS taxonomy to distinguish sports to more precisely investigate their relation with psychologically relevant variables
Source: PLoS One. 2020 Oct 22;15(10):e0241013. doi: 10.1371/journal.pone.0241013 (PMC7581009; doi:10.1371/journal.pone.0241013)
Supplement: S4 Table — (PDF) [file pone.0241013.s004.pdf]

S4 Table. Cluster numbers for the sports for both types of cluster analyses: hierarchical and K-means-cluster analysis

Sport	Cluster in hierarchical cluster analysis	Cluster in *K*-Means cluster analysis
(Half-) Marathon	7	1
Aerobics	9	2
Aikido	12	3
Air hockey	12	3
Air sports (planes)	6	3
Alpine skiing	7	4
Archery	6	4
Artistic gymnastics	3	2
Australian football & Gaelic football (International rules football)	-	5
Auto racing	13	5
Badminton	11	6
Ballet	3	2
Baseball & Softball	16	5
Basketball	16	5
Beach volleyball	-	2
Belly dance	2	2
BMX	2	7
Bodybuilding	1	2
Bodyweight exercises	7	1
Bouldering	4	4
Boules	16	6
Bowling	6	4
Boxing	12	3
Brazilian jiu-jitsu	12	3
Breakdancing	-	2
Calisthenics	7	1
Canoe polo	16	5

Canoeing	2	4
Canyoning	-	4
Capoeira	-	5
Cheerleading	-	2
Chess	-	6
Climbing (outdoor)	4	4
Contemporary dance	9	2
Cricket	13	5
Cross-country cycling & Mountain biking	2	7
Cross-country skiing	7	7
CrossFit	7	2
Cue sports	-	3
Curling	-	5
Dancing	8	2
Darts	5	6
Disc golf	5	4
Dodgeball & Prisonball	-	5
Dragon boat	9	2
Equestrian vaulting	9	2
eSports	13	5
Fencing	12	3
Field hockey	16	5
Fighting sport - Grappling (Other)	12	3
Figure skating	3	2
Flag football	16	5
Floorball	16	5
Freediving	4	4
Golf	10	6
Gridiron football (including American football)	-	5
Handball	16	5
Health club training	1	1
Historical European martial arts	12	3
Horseback riding	6	2

Hurling & Shinty	16	5
Ice hockey	-	5
Indoor climbing	4	4
Indoor cycling	7	1
Indoor soccer	-	6
Inline skating	2	7
Jiujitsu	12	3
Judo	12	3
Jugger	-	5
Karate	12	3
Kendo	12	3
Kickboxing	12	3
Kiteboarding	2	7
Krav Maga	12	3
Kung fu	12	3
Lacrosse	16	5
Longboarding	2	7
Mixed martial arts	12	3
Motocross	5	4
Mountaineering & Hiking	4	4
Obstacle racing	7	4
Paintball & Airsoft	-	5
Paragliding & Hang gliding	6	4
Parkour	2	7
Partner dance	8	2
Pilates	-	1
Pole dance	2	7
Pole vault	6	3
Polo	-	5
Qigong	-	1
Quidditch	15	5
Racewalking	6	6
Rafting & Kayaking	4	4
Recreational cycling	2	7

Road bicycle racing	-	6
Roller derby	-	5
Rowing	-	2
Rugby	16	5
Running	7	1
Sailing	6	4
Shooting sport	6	4
Skateboarding	2	7
Skydiving	4	4
Slacklining	2	7
Snowboarding & Sandboarding	2	7
Soccer	16	5
Sport fishing	5	6
Sprinting	7	2
Squash & Racquetball	11	6
Surfing	2	7
Swimming	7	2
Swordsmanship	12	3
Synchronized swimming	9	5
Table football	10	6
Table tennis	10	6
Tae Bo	9	1
Taekwondo	12	3
Tai chi	-	4
Tennis	10	6
Touch & Tag rugby	-	5
Track and field (combined)	7	2
Track and field (throwing)	6	4
Track cycling	10	6
Trailrunning	7	1
Trampolining	4	4
Triathlon	7	1
Ultimate	15	5
Underwater diving	4	4

Underwater hockey	14	5
Underwater rugby	14	5
Volleyball	16	5
Water polo	16	5
Water skiing	2	2
Weightlifting	7	2
Windsurfing	2	7
Wrestling	-	3
Yoga	2	1
Zumba	2	7
