# Supplementary material for: Using the situational characteristics of the DIAMONDS taxonomy to distinguish sports to more precisely investigate their relation with psychologically relevant variables
Source: PLoS One. 2020 Oct 22;15(10):e0241013. doi: 10.1371/journal.pone.0241013 (PMC7581009; doi:10.1371/journal.pone.0241013)
Supplement: S1 Fig — Sports are sorted alphabetically. The midpoint of the webs reflects a mean value of 1 and the outer line a value of 7. The values for the situational characteristics of the sports are depicted in black. The values for the mean sport situations for the S8* are depicted in green. Standard errors are depicted in dotted lines. (PDF) [file pone.0241013.s005.pdf]

(Half-) Marathon

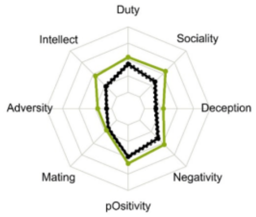

Aerobics

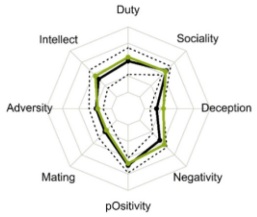

Aikido

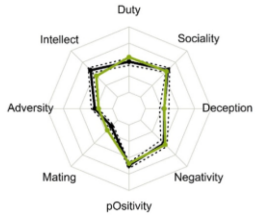

Air hockey

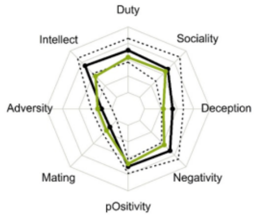

Air sports (planes)

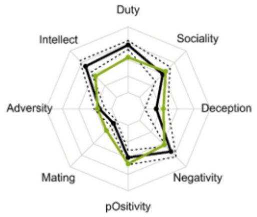

Alpine skiing

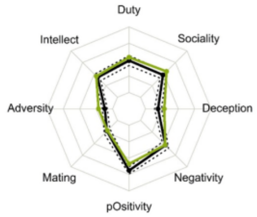

Archery

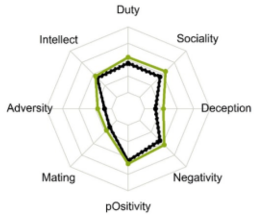

Artistic gymnastics

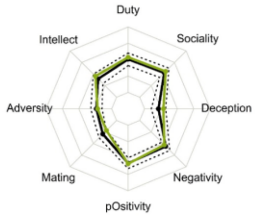

Australian football & Gaelic football (International rules football)

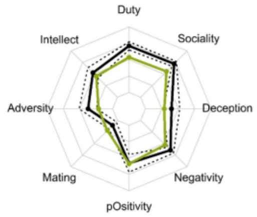

Auto racing

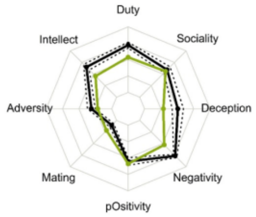

Badminton

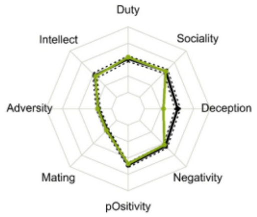

Ballet

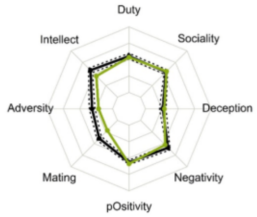

Baseball & Softball

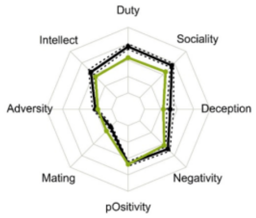

Basketball

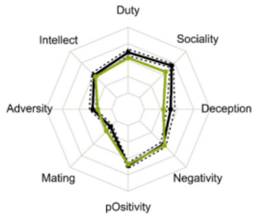

Beach volleyball

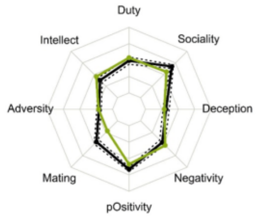

Belly dance

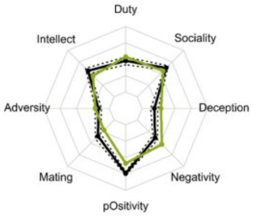

BMX

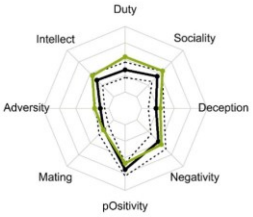

Bodybuilding

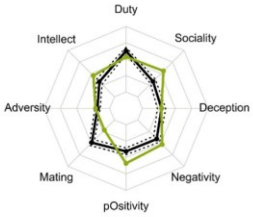

Bodyweight exercises

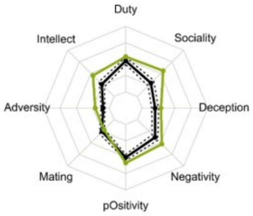

Bouldering

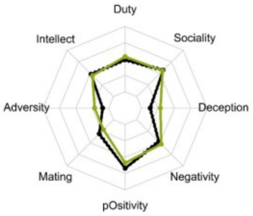

Boules

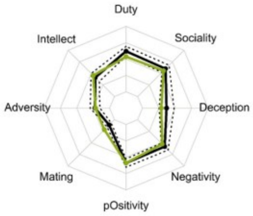

Bowling

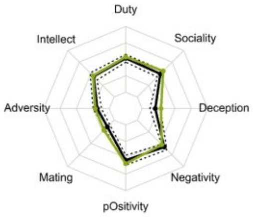

Boxing

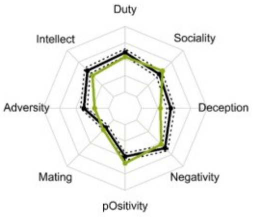

Brazilian jiu-jitsu

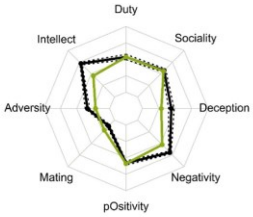

Breakdancing

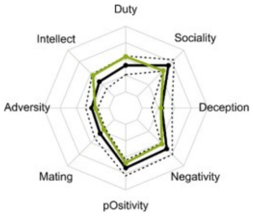

Calisthenics

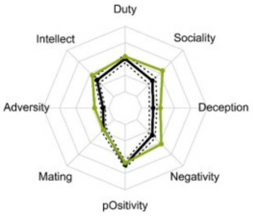

Canoe polo

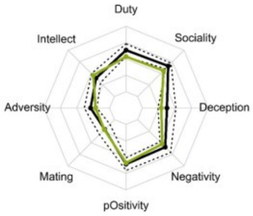

Canoeing

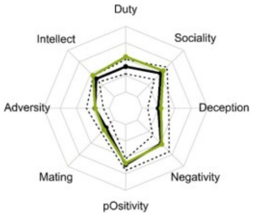

Canyoning

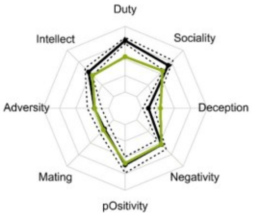

Capoeira

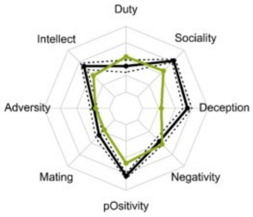

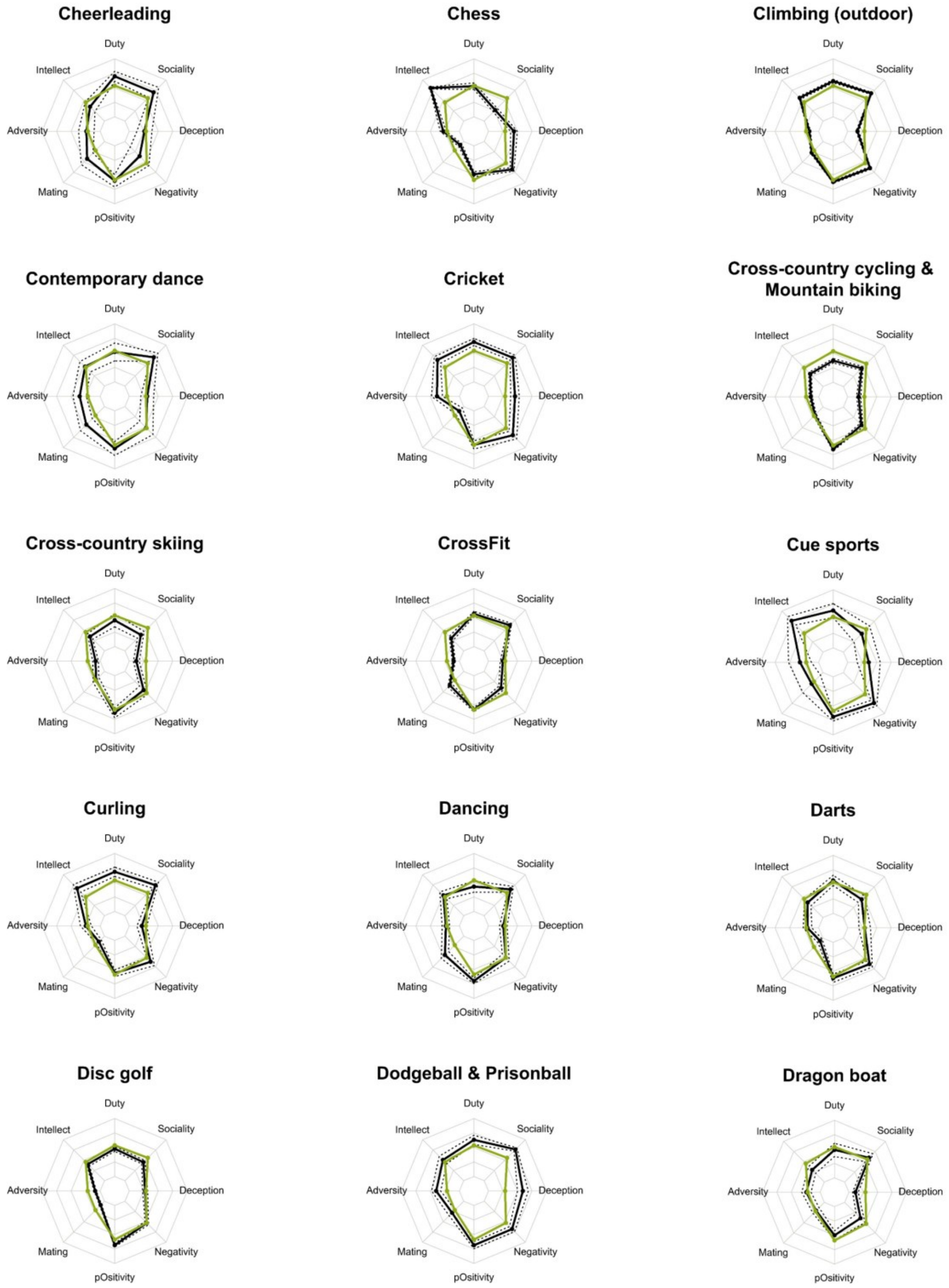
Cheerleading
Duty
Sociality
Deception
Negativity
pOsitivity
Mating
Adversity
Intellect
Chess
Duty
Sociality
Deception
Negativity
pOsitivity
Mating
Adversity
Intellect
Climbing (outdoor)
Duty
Sociality
Deception
Negativity
pOsitivity
Mating
Adversity
Intellect
Contemporary dance
Duty
Sociality
Deception
Negativity
pOsitivity
Mating
Adversity
Intellect
Cricket
Duty
Sociality
Deception
Negativity
pOsitivity
Mating
Adversity
Intellect
Cross-country cycling & Mountain biking
Duty
Sociality
Deception
Negativity
pOsitivity
Mating
Adversity
Intellect
Cross-country skiing
Duty
Sociality
Deception
Negativity
pOsitivity
Mating
Adversity
Intellect
CrossFit
Duty
Sociality
Deception
Negativity
pOsitivity
Mating
Adversity
Intellect
Cue sports
Duty
Sociality
Deception
Negativity
pOsitivity
Mating
Adversity
Intellect
Curling
Duty
Sociality
Deception
Negativity
pOsitivity
Mating
Adversity
Intellect
Dancing
Duty
Sociality
Deception
Negativity
pOsitivity
Mating
Adversity
Intellect
Darts
Duty
Sociality
Deception
Negativity
pOsitivity
Mating
Adversity
Intellect
Disc golf
Duty
Sociality
Deception
Negativity
pOsitivity
Mating
Adversity
Intellect
Dodgeball & Prisonball
Duty
Sociality
Deception
Negativity
pOsitivity
Mating
Adversity
Intellect
Dragon boat
Duty
Sociality
Deception
Negativity
pOsitivity
Mating
Adversity
Intellect

Equestrian vaulting

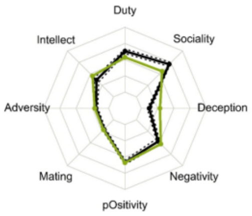

eSports

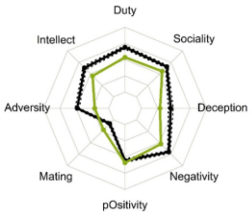

Fencing

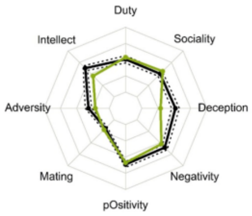

Field hockey

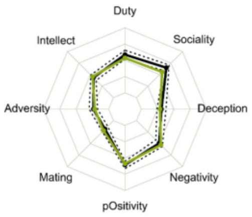

Fighting sport - Grappling (Other)

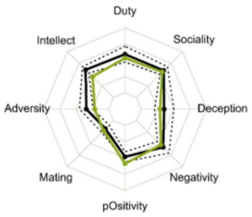

Figure skating

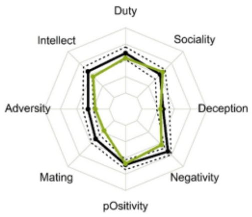

Flag football

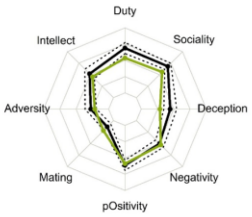

Floorball

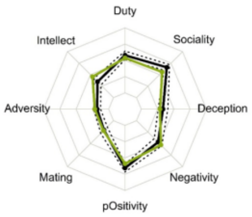

Freediving

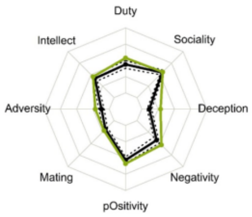

Golf

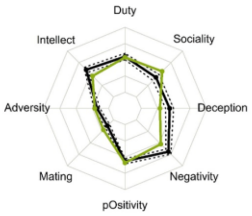

Gridiron football (including American football)

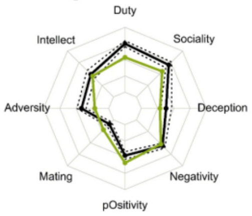

Handball

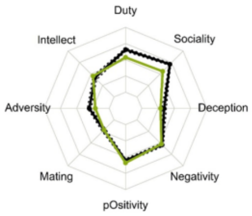

Health club training

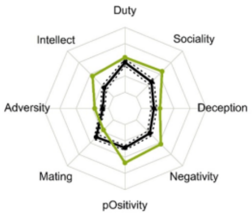

Historical European martial arts

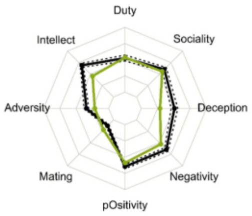

Horseback riding

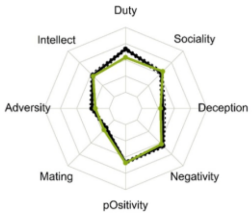

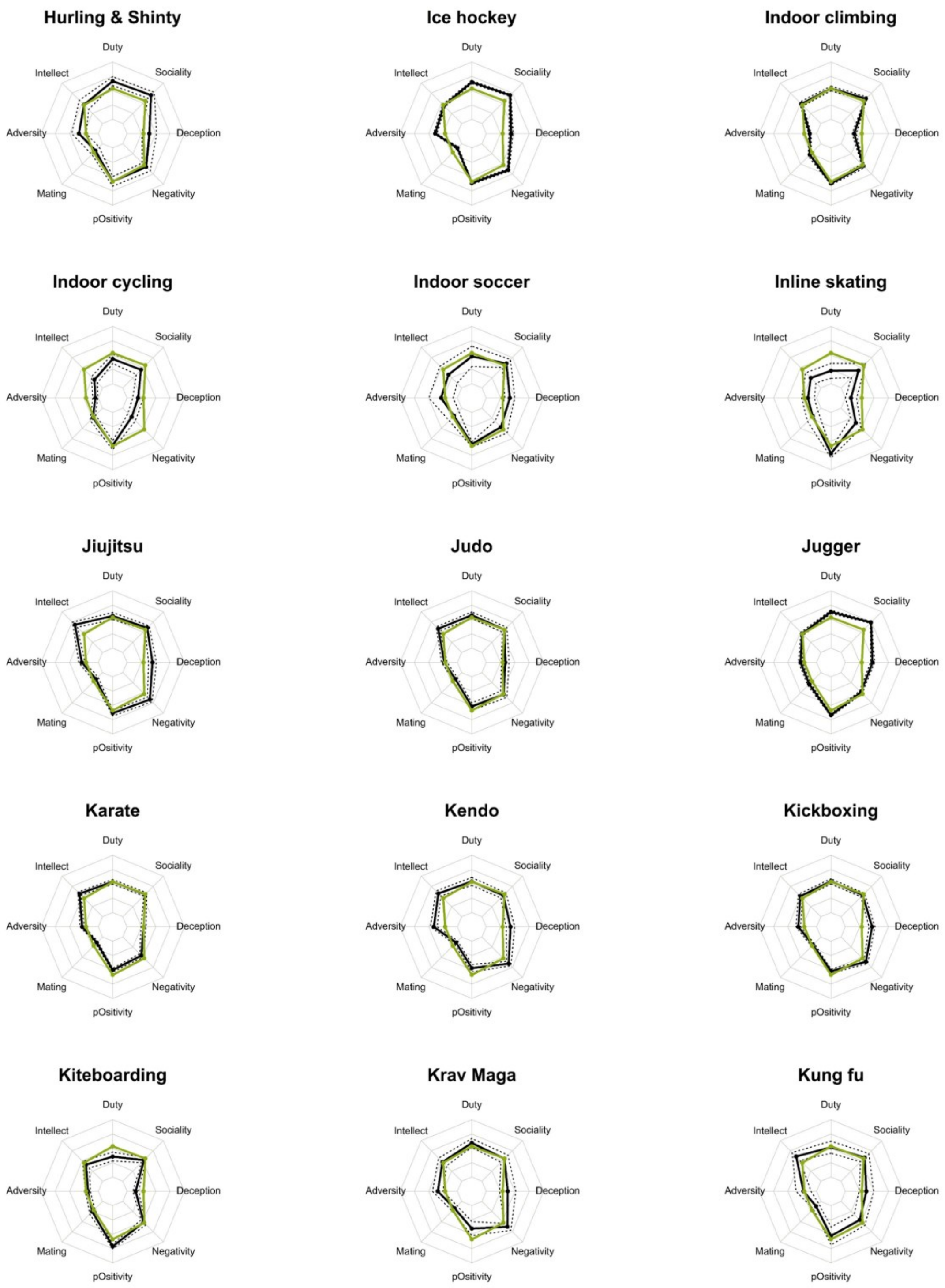
Hurling & Shinty
Duty
Sociality
Deception
Negativity
pOsitivity
Mating
Adversity
Intellect
Ice hockey
Duty
Sociality
Deception
Negativity
pOsitivity
Mating
Adversity
Intellect
Indoor climbing
Duty
Sociality
Deception
Negativity
pOsitivity
Mating
Adversity
Intellect
Indoor cycling
Duty
Sociality
Deception
Negativity
pOsitivity
Mating
Adversity
Intellect
Indoor soccer
Duty
Sociality
Deception
Negativity
pOsitivity
Mating
Adversity
Intellect
Inline skating
Duty
Sociality
Deception
Negativity
pOsitivity
Mating
Adversity
Intellect
Jiujitsu
Duty
Sociality
Deception
Negativity
pOsitivity
Mating
Adversity
Intellect
Judo
Duty
Sociality
Deception
Negativity
pOsitivity
Mating
Adversity
Intellect
Jugger
Duty
Sociality
Deception
Negativity
pOsitivity
Mating
Adversity
Intellect
Karate
Duty
Sociality
Deception
Negativity
pOsitivity
Mating
Adversity
Intellect
Kendo
Duty
Sociality
Deception
Negativity
pOsitivity
Mating
Adversity
Intellect
Kickboxing
Duty
Sociality
Deception
Negativity
pOsitivity
Mating
Adversity
Intellect
Kiteboarding
Duty
Sociality
Deception
Negativity
pOsitivity
Mating
Adversity
Intellect
Krav Maga
Duty
Sociality
Deception
Negativity
pOsitivity
Mating
Adversity
Intellect
Kung fu
Duty
Sociality
Deception
Negativity
pOsitivity
Mating
Adversity
Intellect

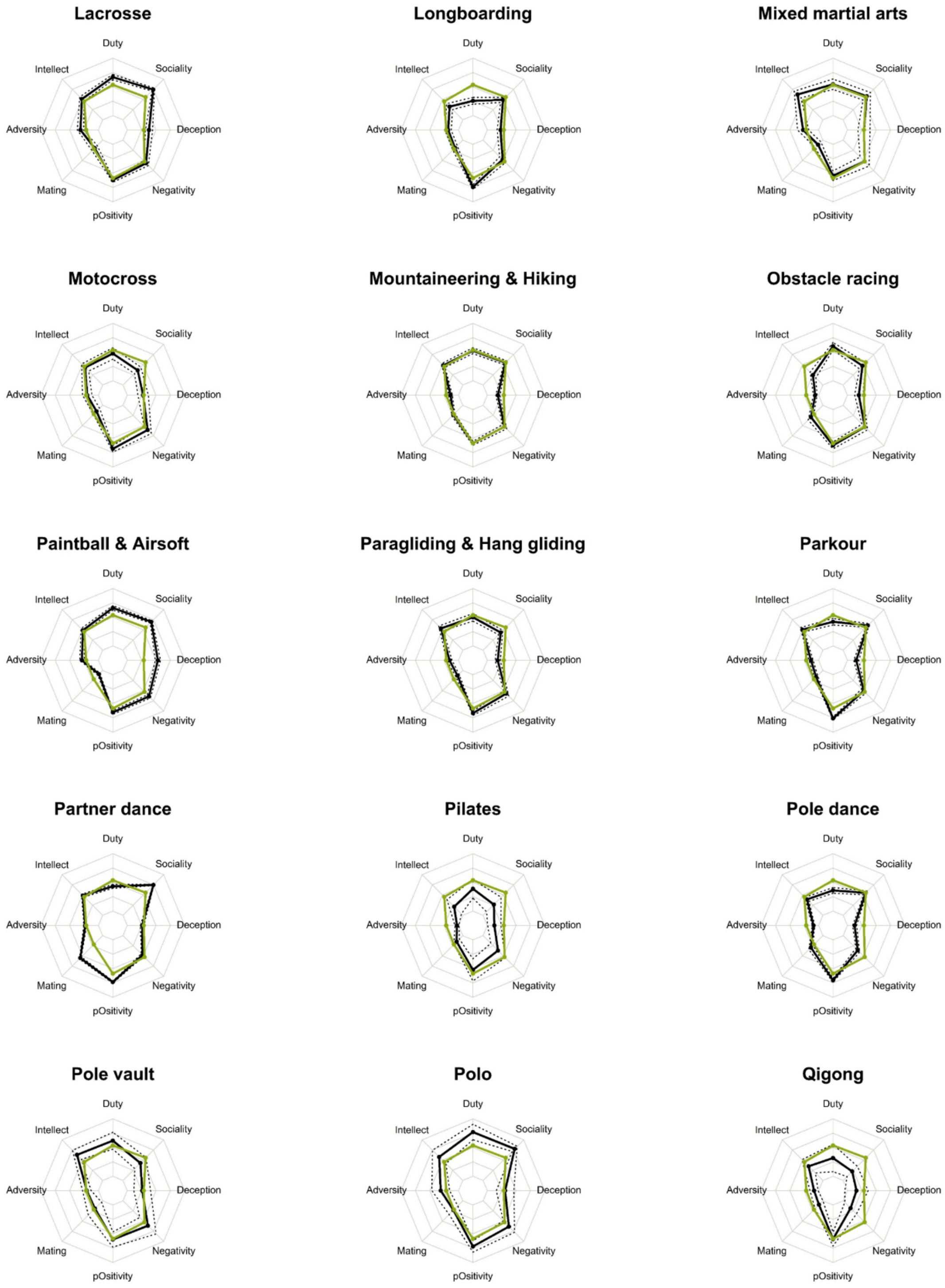
Lacrosse
Longboarding
Mixed martial arts
Motocross
Mountaineering & Hiking
Obstacle racing
Paintball & Airsoft
Paragliding & Hang gliding
Parkour
Partner dance
Pilates
Pole dance
Pole vault
Polo
Qigong
Duty
Sociality
Deception
Negativity
pOsitivity
Mating
Adversity
Intellect

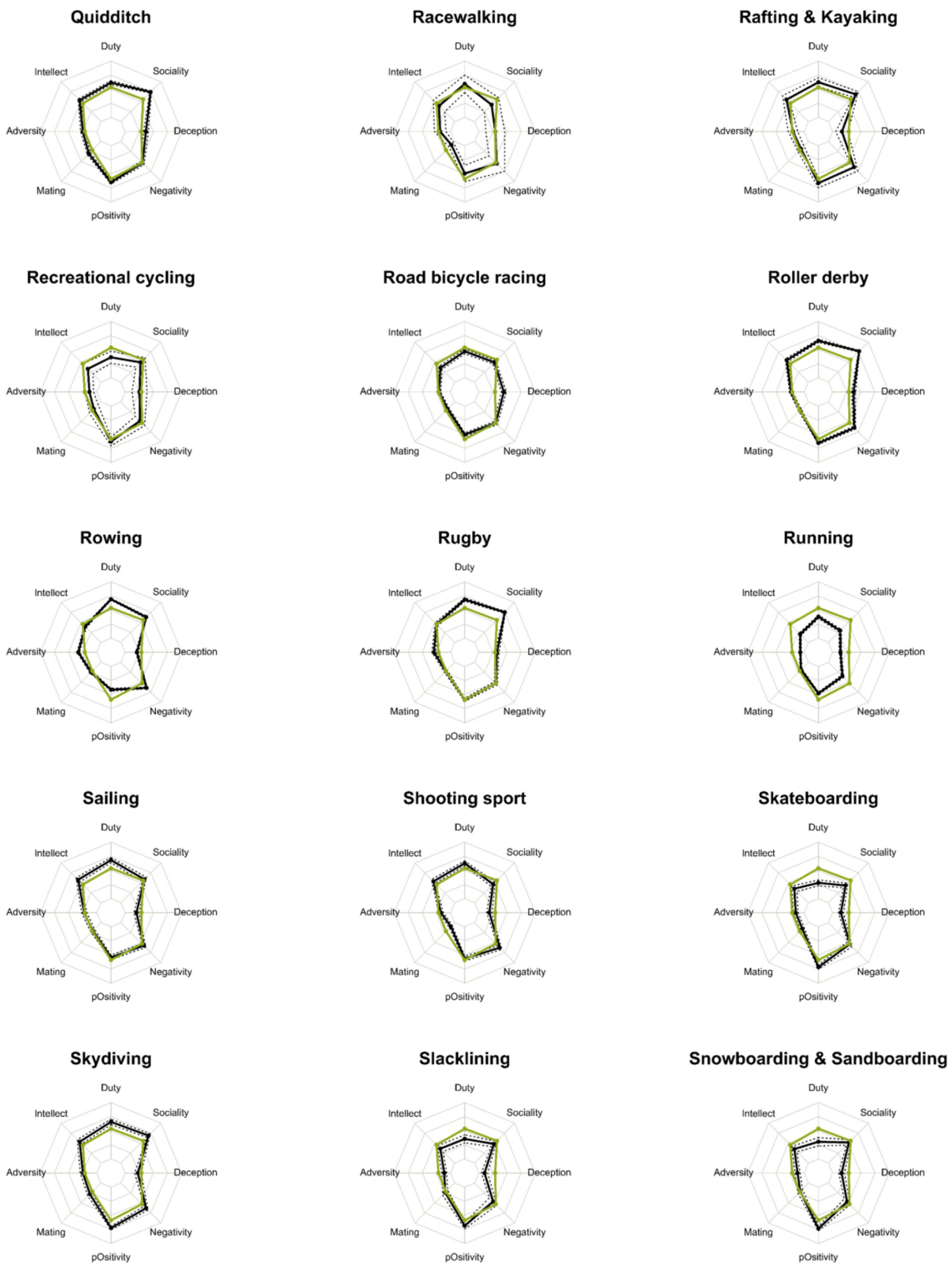
Quidditch
Racewalking
Rafting & Kayaking
Recreational cycling
Road bicycle racing
Roller derby
Rowing
Rugby
Running
Sailing
Shooting sport
Skateboarding
Skydiving
Slacklining
Snowboarding & Sandboarding
Duty
Sociality
Deception
Negativity
pOsitivity
Mating
Adversity
Intellect

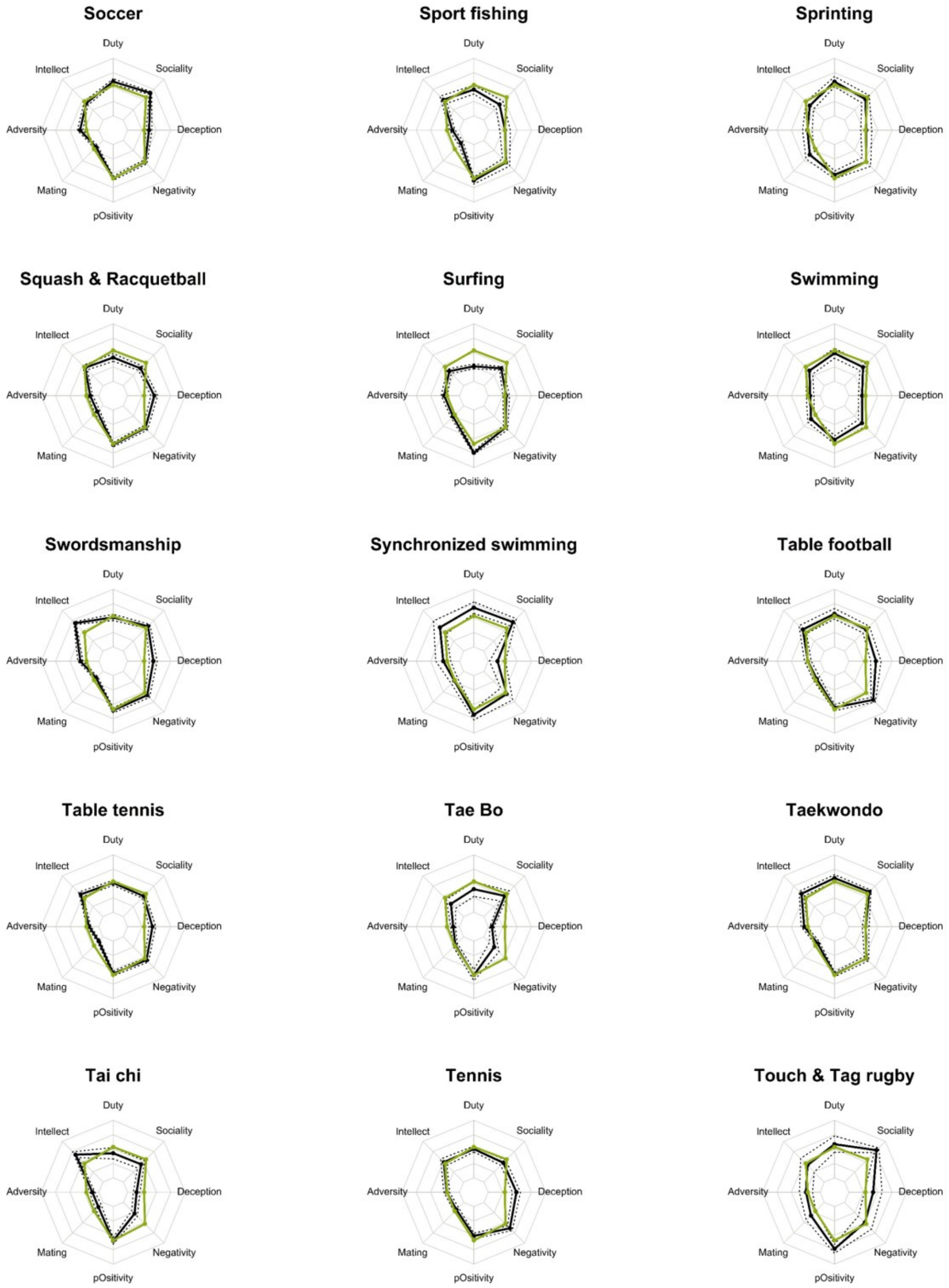
Soccer
Duty
Sociality
Deception
Negativity
pOsitivity
Mating
Adversity
Intellect
Sport fishing
Duty
Sociality
Deception
Negativity
pOsitivity
Mating
Adversity
Intellect
Sprinting
Duty
Sociality
Deception
Negativity
pOsitivity
Mating
Adversity
Intellect
Squash & Racquetball
Duty
Sociality
Deception
Negativity
pOsitivity
Mating
Adversity
Intellect
Surfing
Duty
Sociality
Deception
Negativity
pOsitivity
Mating
Adversity
Intellect
Swimming
Duty
Sociality
Deception
Negativity
pOsitivity
Mating
Adversity
Intellect
Swordsmanship
Duty
Sociality
Deception
Negativity
pOsitivity
Mating
Adversity
Intellect
Synchronized swimming
Duty
Sociality
Deception
Negativity
pOsitivity
Mating
Adversity
Intellect
Table football
Duty
Sociality
Deception
Negativity
pOsitivity
Mating
Adversity
Intellect
Table tennis
Duty
Sociality
Deception
Negativity
pOsitivity
Mating
Adversity
Intellect
Tae Bo
Duty
Sociality
Deception
Negativity
pOsitivity
Mating
Adversity
Intellect
Taekwondo
Duty
Sociality
Deception
Negativity
pOsitivity
Mating
Adversity
Intellect
Tai chi
Duty
Sociality
Deception
Negativity
pOsitivity
Mating
Adversity
Intellect
Tennis
Duty
Sociality
Deception
Negativity
pOsitivity
Mating
Adversity
Intellect
Touch & Tag rugby
Duty
Sociality
Deception
Negativity
pOsitivity
Mating
Adversity
Intellect

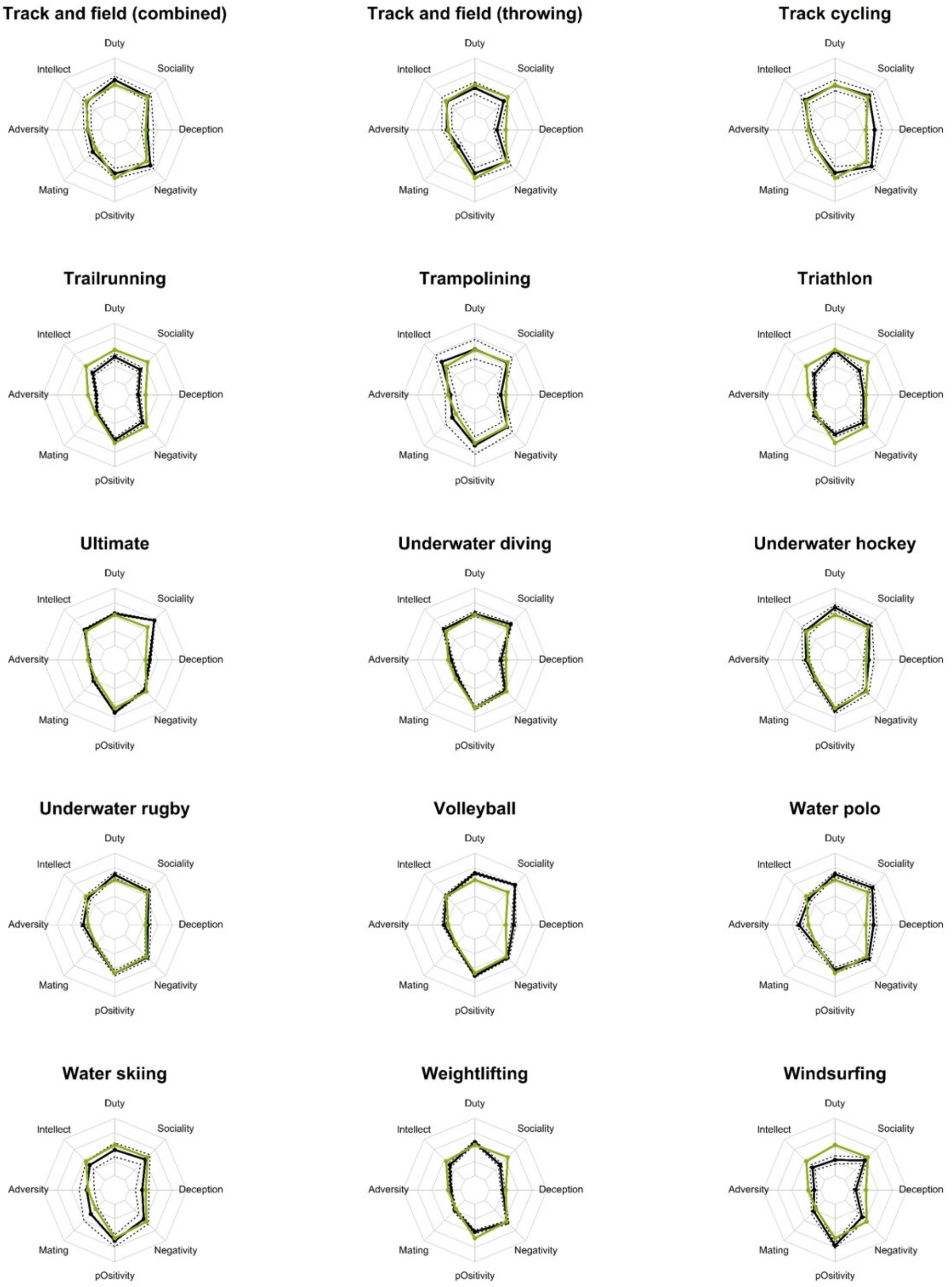
Track and field (combined)
Duty
Sociality
Deception
Negativity
pOsitivity
Mating
Adversity
Intellect
Track and field (throwing)
Duty
Sociality
Deception
Negativity
pOsitivity
Mating
Adversity
Intellect
Track cycling
Duty
Sociality
Deception
Negativity
pOsitivity
Mating
Adversity
Intellect
Trailrunning
Duty
Sociality
Deception
Negativity
pOsitivity
Mating
Adversity
Intellect
Trampolining
Duty
Sociality
Deception
Negativity
pOsitivity
Mating
Adversity
Intellect
Triathlon
Duty
Sociality
Deception
Negativity
pOsitivity
Mating
Adversity
Intellect
Ultimate
Duty
Sociality
Deception
Negativity
pOsitivity
Mating
Adversity
Intellect
Underwater diving
Duty
Sociality
Deception
Negativity
pOsitivity
Mating
Adversity
Intellect
Underwater hockey
Duty
Sociality
Deception
Negativity
pOsitivity
Mating
Adversity
Intellect
Underwater rugby
Duty
Sociality
Deception
Negativity
pOsitivity
Mating
Adversity
Intellect
Volleyball
Duty
Sociality
Deception
Negativity
pOsitivity
Mating
Adversity
Intellect
Water polo
Duty
Sociality
Deception
Negativity
pOsitivity
Mating
Adversity
Intellect
Water skiing
Duty
Sociality
Deception
Negativity
pOsitivity
Mating
Adversity
Intellect
Weightlifting
Duty
Sociality
Deception
Negativity
pOsitivity
Mating
Adversity
Intellect
Windsurfing
Duty
Sociality
Deception
Negativity
pOsitivity
Mating
Adversity
Intellect

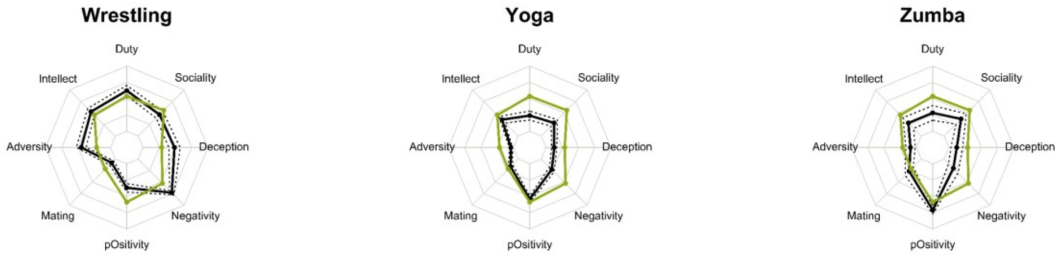

S1 Fig. Graphical presentation of the mean values (M) and standard errors (SD) for each of the eight DIAMONDS scales for each Sport. Sports are sorted alphabetically. The midpoint of the webs reflects a mean value of 1 and the outer line a value of 7. The values for the situational characteristics of the sports are depicted in black. The values for the mean sport situations for the S8* are depicted in green. Standard errors are depicted in dotted lines.
