# Supplementary material for: Using the situational characteristics of the DIAMONDS taxonomy to distinguish sports to more precisely investigate their relation with psychologically relevant variables
Source: PLoS One. 2020 Oct 22;15(10):e0241013. doi: 10.1371/journal.pone.0241013 (PMC7581009; doi:10.1371/journal.pone.0241013)
Supplement: S2 Fig — Clusters framed in red depict clusters with p-values > .95 for 1000 bootstrap repetitions. (PDF) [file pone.0241013.s006.pdf]

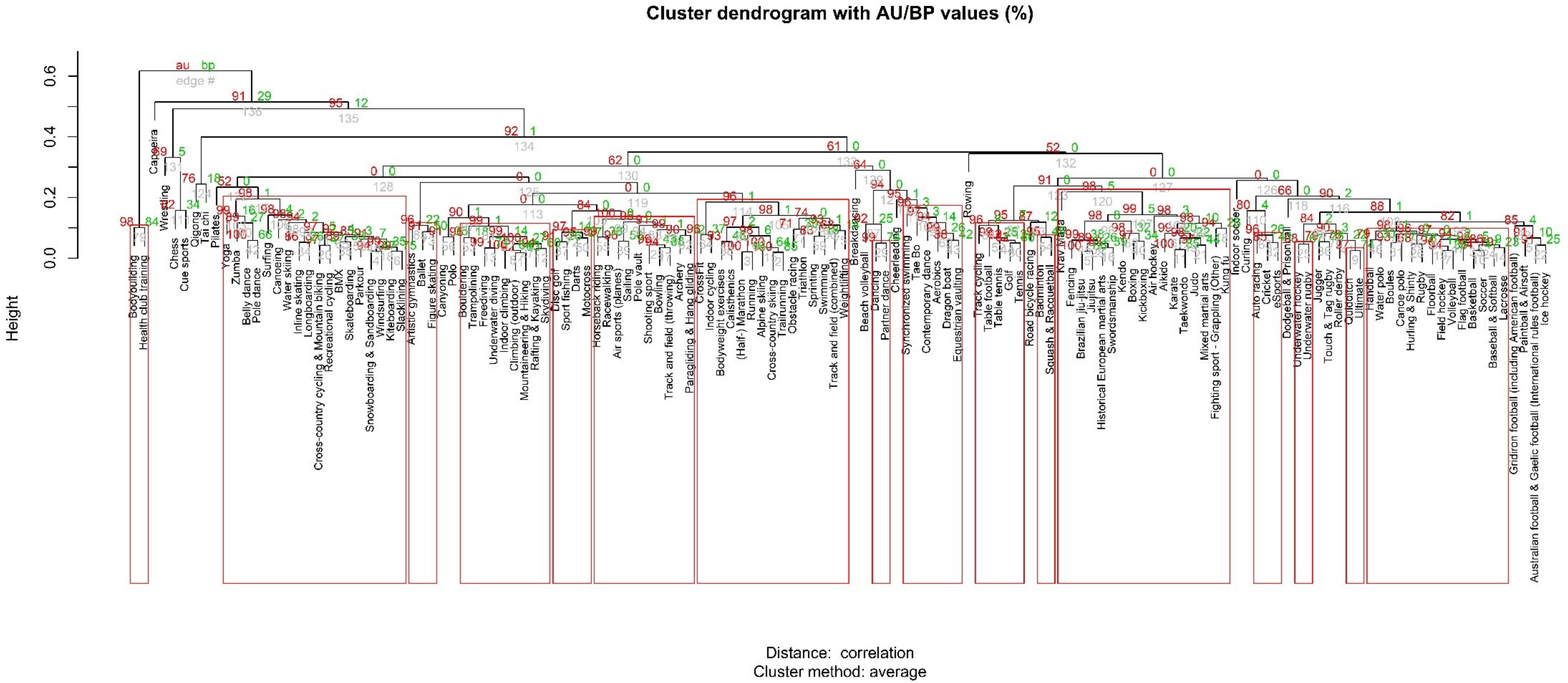

S2 Fig. Result of the hierarchical agglomerative cluster analysis for the overall similarity comparison. Clusters framed in red depict clusters with p-values > .95 for 1000 bootstrap repetitions.
